# Attributing climate-related health burdens in Southeast Asia: a scoping review

**DOI:** 10.1016/j.lanwpc.2026.101952

**Published:** 2026-08-21

**Authors:** Robin J. Simion, Min Thu, Perla Boutros, Syahrul Nellis, Julia Feriato Corvetto, Tin Tin Su, Sandra Barteit

**Affiliations:** a Heidelberg Institute of Global Health (HIGH), Faculty of Medicine and University Hospital, Heidelberg University, Heidelberg, Germany; b South East Asia Community Observatory (SEACO), Global Population Health, Jeffrey Cheah School of Medicine & Health Sciences, Monash University Malaysia, Bandar Sunway, 47500, Malaysia

**Keywords:** Global health, Cliamte change, Attribution, Impact attribution, Southeast Asia, Western Pacific, Health, Heat, Cold, Extreme events, Temperature, Temperature variability, Mortality, Morbidity, Attributable fraction, Populations attributable fraction

## Abstract

Attributing health outcomes to climate-related exposures is essential for informing adaptive policy, yet Southeast Asia, one of the world's most climate-vulnerable regions, remains critically underrepresented in attribution research. To address this gap, we conducted a scoping review of studies linking climate exposures to health burdens in the region. Although growing evidence demonstrates substantial health impacts from air pollution, heat, and extreme weather, the regional research landscape remains critically imbalanced, marked by neglect of other major hazards—including floods, droughts, extreme rainfall, storms and sea-level rise—and substantial methodological fragmentation. From 5294 records screened, 77 studies met our inclusion criteria. The evidence is dominated by air pollution research, the focus of 70% of studies and linked to up to 31% of all-cause mortality and an estimated 296,000 annual deaths from wildfire smoke during El Niño events. Heat, the second most studied exposure, was associated with up to 8.61% of mortality in Bangkok. In contrast, extreme events such as heatwaves, floods and storms were least frequently investigated. These findings reveal a critical misalignment between regional climate risks and current research focus. Standardized attribution frameworks are urgently needed to support integrated climate-health modeling and guide equitable, evidence-based adaptation across Southeast Asia.

## Introduction

The health impacts of climate change are increasingly evident globally. Rising temperatures are contributing to a higher frequency and intensity of extreme weather events, deteriorating air quality, and a cascade of associated health risks.^1^, ^2^, ^3^, ^4^, ^5^, ^6^, ^7^, ^8^, ^9^ Although epidemiological methods have long been used to quantify disease burdens from environmental exposures, the systematic integration of these approaches within the formal framework of climate attribution science remains methodologically and regionally uneven.^10^

Attribution studies, which estimate the likelihood of climate-related events under anthropogenic versus natural conditions, have become crucial tools for understanding the role of human-induced climate change.^11^^,^^12^ When linked with epidemiological data, these methods allow for the estimation of the burden of disease directly attributable to climate change. Such health attribution studies are essential for informing equitable adaptation strategies and have become central to global discussions around climate-related loss and damages, particularly since the 27th United Nations Climate Change Conference (COP27).^13^

However, the current climate–health attribution literature is characterized by a pronounced geographical imbalance. Low- and middle-income countries (LMICs) remain substantially underrepresented, despite bearing a disproportionate burden of climate-sensitive health risks.^14^ Analysis of publications indexed in Web of Science indicates that Southeast Asia accounts for only approximately 4.5% of global climate–health research output based on author affiliations, despite comprising around 8.5% of the global population (approximately 680 million people). This disparity underscores a marked underrepresentation of the region in the evidence base. The imbalance is particularly concerning given that populations in Southeast Asia are exposed to multiple, compounding climate-related hazards, including rising temperatures, high humidity, worsening air pollution, and an increasing frequency of extreme weather events.^15^ These environmental risks intersect with a triple disease burden—comprising communicable diseases, non-communicable conditions, and climate change challenges—which exacerbates regional health vulnerabilities.^16^ Despite these factors, there is a profound lack of systematic quantification of the health outcomes attributable to climate-related exposures in Southeast Asia.

This underrepresentation is also evident in the global evidence base used to derive exposure–response functions for climate–health burden estimation. Recent systematic reviews of long-term particulate matter exposure and mortality in the Asia–Pacific region identified cohort evidence from Australia, mainland China, Hong Kong, Taiwan, South Korea, and Japan, but no cohort studies from Southeast Asian countries.^17^^,^^18^ Similarly, global reviews of long-term particulate matter exposure and mortality remain dominated by studies from North America and Europe, with the comparatively small Asian evidence base largely originating from East Asia.^19^ This imbalance has direct methodological implications for attribution research, because widely used pooled exposure–response functions, including the Global Exposure Mortality Model, are derived from cohort data with limited Asian and effectively no Southeast Asian population representation.^18^ Importantly, this evidence gap is not uniform across all climate-sensitive health outcomes: meta-analyses of temperature-sensitive infectious diseases, such as dengue, include a larger contribution from Southeast Asian and other low- and middle-income settings.^20^ The underrepresentation is therefore exposure- and outcome-specific, and is most pronounced in the temperature- and air-pollution-mortality literature that currently forms a central basis for climate–health attribution estimates.

This knowledge gap is perpetuated by both methodological fragmentation and multiple structural barriers.^21^^,^^22^ Methodologically, the existing literature is difficult to synthesize; country-specific studies, particularly from Thailand and Vietnam, often focus narrowly on urban settings and employ heterogeneous definitions, attribution metrics, and analytical designs.^23^, ^24^, ^25^ This inconsistency makes cross-country comparisons difficult and limits the generalizability of findings for regional policy. Moreover, few studies have assessed the joint effects of multiple exposures, such as heat and air pollution, despite their known synergistic impacts. These methodological issues are compounded by structural barriers, including sparse observational health and environmental datasets, restricted access to regional climate models (RCMs), and limited local technical capacity to conduct integrative modeling.^26^^,^^27^

Furthermore, previous global syntheses have not adequately captured the regional context of Southeast Asia. The Wellcome Trust's 2021 global review, for instance, applied strict inclusion criteria requiring direct attribution to anthropogenic climate change. This standard resulted in the exclusion of a large body of exposure-based literature highly relevant to the Southeast Asian context, including crucial studies on ambient air pollution and temperature variability.^12^ As a result, significant exposure domains and large parts of the global South remain underrepresented in our current understanding, while the field's methodological heterogeneity continues to hinder cross-study comparisons and integration into climate policy frameworks.^28^, ^29^, ^30^

This scoping review addresses these multifaceted limitations by systematically synthesizing attribution studies that link climate-related exposures to health outcomes across Southeast Asia. It aims to provide the first comprehensive overview of the regional evidence base, identify underexamined exposure–health relationships, and critically evaluate the consistency and comparability of the attribution methodologies used. By delineating these key empirical and methodological gaps, this review supports the development of standardized climate-health attribution frameworks and informs future efforts in regional disease burden modeling, climate adaptation policy, and equity-oriented health planning.

## Methods

To address the knowledge gaps surrounding the health impacts of climate change, we conducted a scoping review. This approach was chosen to systematically map the emerging research on impact attribution, identify key theoretical frameworks and evidence sources, and define the boundaries of the current peer-reviewed literature. The review was performed in accordance with the PRISMA Extension for Scoping Reviews (PRISMA-ScR),^31^ with the full protocol available in the Supplementary Material (Figure S1).

### Search strategy

We performed a systematic literature search of four databases: PubMed (Medline), Scopus, Embase, and Web of Science. The search was last updated in April 2026.

Our search strategy was constructed around four core concepts linked with Boolean operators: (i) Southeast Asian (SEA) countries, (ii) climate exposures, (iii) health outcomes, and (iv) attribution. Search strings were adapted to the specifications of each database, such as using MeSH terms in PubMed. The complete search strings are provided in the Supplementary Material.

### Selection criteria

Studies were eligible for inclusion if they met the following criteria:

• **Publication Type:** The study had to be original, peer-reviewed, primary research published in English.
• **Subject:** The research focused on human subjects.
• **Core requirement:** Studies were required to quantify the health burden attributable to a primary climate, weather, or environmental exposure using a formal attributable-burden metric, including attributable fraction (AF), attributable number (AN), population attributable fraction (PAF), and population attributable number (PAN), or a mathematically equivalent counterfactual estimate. Studies reporting only measures of association without reference to a counterfactual, for example incidence rate ratios, relative risks, or percentage change per unit of exposure, were not eligible on the basis of association alone. Qualitative attribution, including narrative or theoretical attribution without quantitative burden estimation, was outside the scope of this review. Attribution metrics were coded according to their mathematical equivalence to AF, AN, PAF, or PAN, rather than the terminology used by the original study.
• **Geographical Scope:** Data originated from Southeast Asian countries including Brunei, Cambodia, Indonesia, Laos, Malaysia, Myanmar, the Philippines, Singapore, Thailand, Timor-Leste, or Vietnam.
• **Eligible Health Outcomes:** Outcomes included mortality (all-cause, cause-specific, non-external, or years of life lost [YLL]); morbidity (e.g., heat-related illness, hospital admissions, or years lived with disability [YLD]); or disease burden indicators (e.g., disability-adjusted life years [DALY]).
• **Eligible Climate Exposures:** Exposures, based on the IPCC Sixth Assessment Report (AR6),^32^ included heat, cold, extreme rainfall, droughts, floods, storms, and non-human-emitted air pollution, provided that they were quantified using primary meteorological data.

Studies were excluded if they did not meet these criteria or if the full text could not be retrieved through open access or institutional access available to the review team.

### Study screening and data extraction

Three reviewers (RSi, MT, JC) independently screened all titles and abstracts using Covidence^33^ software. The full texts of potentially relevant studies were then reviewed against the inclusion criteria by three reviewers (RSi, PB, MT). Any discrepancies were resolved through discussion.

Data extraction was performed by two authors (RSi, PB) using a customized template in Covidence's data extraction tool. The extracted data were processed in Microsoft Excel (version 16.93.1). Visualizations were generated using Excel and Python (version 3.11) with the pandas, numpy, matplotlib, and seaborn libraries.

## Results

### Study selection and characteristics

**Fig. 1:**
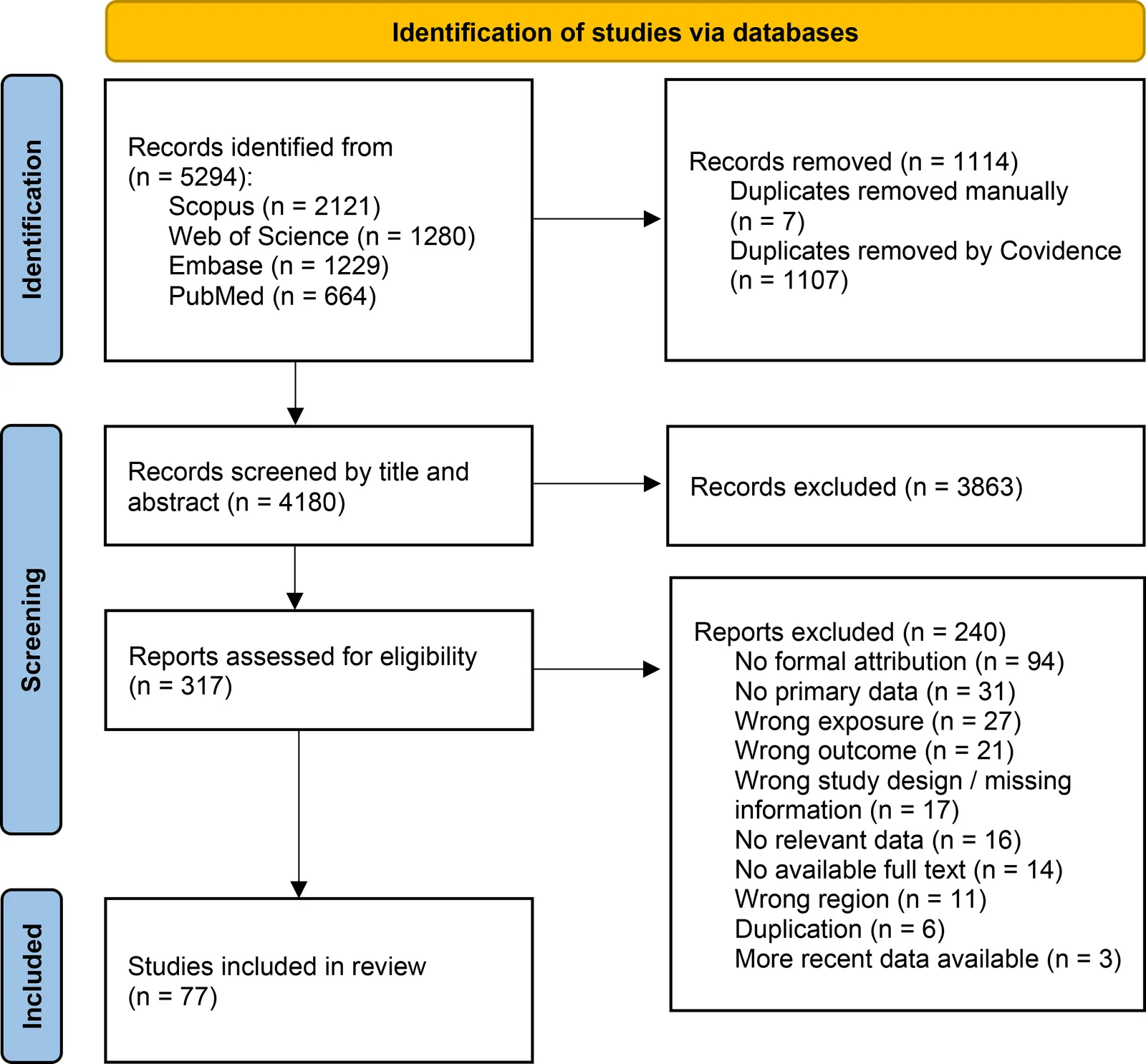
PRISMA-ScR flow diagram outlining the systematic process of study identification, screening, eligibility assessment, and inclusion, with detailed counts of included and excluded records at each stage.

A systematic search across four major bibliographic databases yielded 5294 records. After removing 1114 duplicates, 4180 unique records were screened by title and abstract. Of these, 3863 were excluded for not meeting inclusion criteria. Full-text review was conducted for 317 articles, resulting in the exclusion of 240 studies due to absence of a formal attribution metric (n = 94), reliance on non-primary meteorological data (n = 31), assessment of ineligible exposure types (n = 27), irrelevant outcomes (n = 21) or other reasons (n = 67). A final set of 77 studies met all eligibility criteria and were included for full data extraction and synthesis (Fig. 1).

### Temporal and geographic distribution

**Fig. 2:**
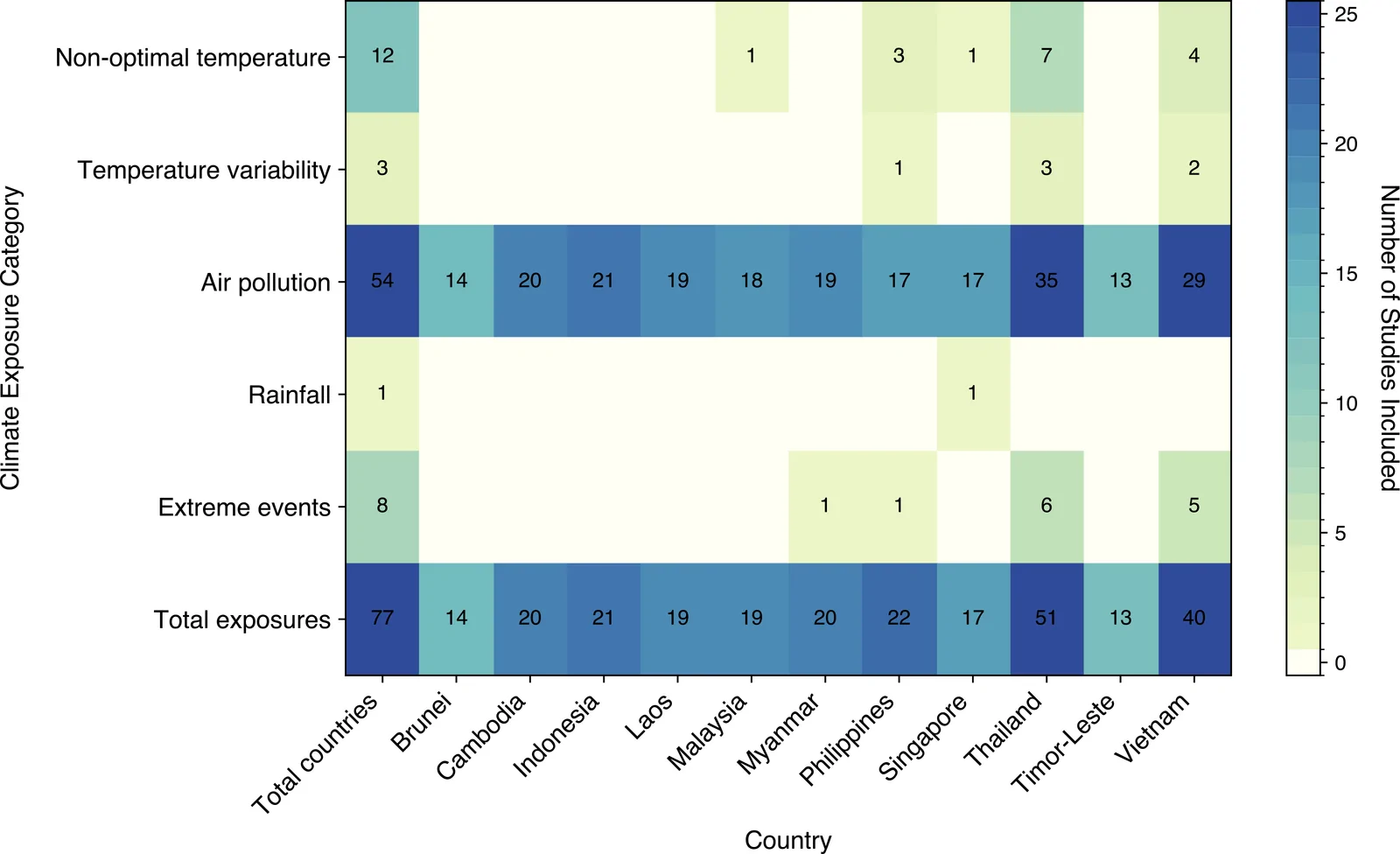
Distribution of climate exposure-health attribution studies in Southeast Asian Countries by exposure type (n = 77). Climate exposures include temperature, air pollutants, extreme events, and rainfall. Studies can present data for multiple countries/exposures.

The volume of published studies on climate–health attribution in Southeast Asia has grown steadily since 2012, with a modest decline in 2024 (Table 1). Research output is concentrated in Thailand (n = 51, 66%) and Vietnam (n = 40, 52%), whereas countries such as Timor-Leste (n = 13, 17%) and Brunei (n = 14, 18%) remain markedly underrepresented, with Timor-Leste appearing exclusively in regional studies and Brunei the subject of only a single country-specific analysis. Intermediate representation was observed in the remaining countries, each contributing between 17, and 22 studies (22–29%) (Fig. 2).

**Table 1:** Methodological characteristics of climate–health attribution studies conducted in Southeast Asia (n = 77).

	n (%)
Study designs (n = 77)	
Time-series	72 (94)
Case-crossover	3 (4)
Case-control	1 (1)
Cohort study	1 (1)
Publication year (n = 77)	
2012	2 (3)
2013	1 (1)
2014	1 (1)
2015	2 (3)
2016	1 (1)
2017	1 (1)
2018	5 (6)
2019	2 (3)
2020	6 (8)
2021	10 (13)
2022	11 (14)
2023	11 (14)
2024	6 (8)
2025	12 (16)
2026	6 (8)
Time periods (n = 77)	
<1 year	4 (5)
1 year	13 (17)
1–10 years	29 (38)
>10 years	31 (40)
Health outcomes (n = 77)	
Mortality	62 (81)
Morbidity	19 (25)
DALYs	3 (4)
YLL	1 (1)
Morbidity outcome measures^a^ (n = 19)	
Hospital admissions	14 (74)
Cases	4 (21)
Emergency department visits	2 (11)
Outpatient department visits	2 (11)
Attributable outcome measures^a^ (n = 77)	
Population attributable number	33 (43)
Attributable fraction	26 (34)
Attributable number	24 (31)
Population attributable fraction	9 (12)
Projections (n = 77)	
No projection	71 (92)
Near future (<2030)	4 (5)
Late future (2100)	2 (3)

Mortality is reported as a single category as it is a binary outcome; morbidity is sub-stratified to reflect the heterogeneity of the underlying outcome measures; DALYs, disability adjusted life years; YLL, year of life lost.

This table summarizes exposure types, health outcomes, and attribution outcome measures, while. Percentages are calculated relative to the total number of studies reporting each parameter.

a Study characteristics can be represented in multiple instances.

### Climate exposures and associated health outcomes

Air pollution was the most frequently assessed exposure, with 70% of studies (n = 54) examining pollutants such as PM_2.5_, PM_10_, and ozone (O_3_). Temperature-related exposures, including non-optimal temperatures (above and/or below the minimum mortality temperature) and temperature variability (fluctuations within or between days), were addressed in 17% (n = 13) and 4% (n = 3) of studies, respectively. By contrast, extreme events such as floods, storms, and heatwaves were investigated in only 10% of studies (n = 8), and rainfall in just 1% (n = 1) (Fig. 2).

The most frequently studied health outcomes were mortality (81%, n = 62) and morbidity (25%, n = 19), with limited attention to broader health burden indicators such as disability-adjusted life years (DALYs) (4%, n = 3) and years of life lost (YLL) (1%, n = 1). Within morbidity-related studies, the most commonly reported metrics were hospital admissions and disease cases, followed by emergency department visits and outpatient department visits (Table 1).

### Methodological approaches and timeframes

The majority of studies (94%, n = 72) used time-series analyses, with only a few employing case-crossover (4%, n = 3), case–control (1%, n = 1) or cohort study designs (1%, n = 1). The study periods were diverse: 38% (n = 29) spanned 1–10 years, 40% (n = 31) extended beyond a decade, while projection-based studies addressed either the near future before 2030 (5%, n = 4) or late-century scenarios to 2100 (3%, n = 2) under varying Representative Concentration Pathway (RCP) scenarios (Table 1).

Health data were primarily derived from national or public databases (45%, n = 35), followed by global datasets such as the Global Burden of Disease (GBD) study (26%, n = 20), the Multi-Country Multi-City (MCC) project (8%, n = 6), and the World Health Organization (WHO) (8%, n = 6). Meteorological exposure data were most commonly derived from satellite-based aerosol optical depth AOD products (35%, n = 27), followed by national or public monitoring networks (34%, n = 26) and atmospheric models (25%, n = 19). Population data—critical for calculating population attributable fractions (PAFs)—were most often sourced from NASA's Gridded Population of the World—Socioeconomic Data and Applications Center (GPW-SEDAC) (27%, n = 21) or from national reports (22%, n = 17), although 34% (n = 26) of studies did not report their population data source (Table 2).

**Table 2:** Methodological characteristics of climate–health attribution studies conducted in Southeast Asia (n = 77).

	n (%)
Health data sources (n = 77)	
National/public networks	35 (45)
GBD database	20 (26)
MCC database	6 (8)
WHO database	6 (8)
Hospitals	3 (4)
Previous study	2 (3)
World Bank	1 (1)
Third party study	1 (1)
Self-collection	1 (1)
Not mentioned	2 (3)
Meteorological data sources (n = 77)	
Satellite derived (AOD)	27 (35)
National/public networks	26 (34)
Atmospheric models	19 (25)
Self-monitored	4 (5)
Not mentioned	1 (1)
Population data sources (n = 77)	
GWP/SEDAC	21 (27)
National/official reports	17 (22)
LandScan	5 (6)
GBD population data	2 (3)
World Bank	2 (3)
Self-collected	2 (3)
MCC database	1 (1)
WorldPop	1 (1)
Not mentioned	26 (34)
Modeling framework|Non-optimal temperature^a^ (n = 13)	
Self-established	13 (100)
Statistical model^a^	
(Quasi-)Poisson	12 (92)
GLM	2 (15)
GAM	1 (8)
Functional form^a^	
Non-linear	9 (69)
Log-linear	4 (31)
Linear	1 (8)
DLNM usage	
Yes	12 (92)
No	1 (8)
Modeling framework|Temperature variability (n = 3)	
Self-established	3 (100)
Statistical model	
(Quasi-)Poisson	3 (100)
Functional form	
Non-linear	2 (67)
Linear	1 (33)
DLNM usage	
Yes	2 (67)
No	1 (33)
Modeling framework^a^|Air pollution (n = 54)	
Self-established function n = 11 (20)	
Statistical model^a^	
(Quasi-)Poisson	7 (64)
Logistic regression	2 (18)
GAM	2 (18)
Cox regression	1 (9)
Not mentioned	1 (9)
Functional form^a^	
Non-linear	6 (55)
Log-linear	5 (45)
Linear	1 (9)
DLNM usage	
Yes	1 (9)
No	10 (91)
ERF source entries|Air pollution (n = 64)	
IER–Burnett (2014)	9 (14)
GEMM–Burnett (2018)	7 (11)
GBD MR-BRT (2019)	4 (6)
CRA (2004)	2 (3)
AirQ+ (2016)	2 (3)
Pope (2011)	2 (3)
Ostro (2004)	2 (3)
Chen & Hoek (2020)	2 (3)
Jerrett (2009)	2 (3)
Vodonos (2018)	2 (3)
Multiple ERF sources (>5)	4 (6)
Other single-entry ERFs	26 (41)
Modeling framework|Rainfall (n = 1)	
Self-established	1 (100)
Statistical model	
GAM + Poisson	1 (100)
Functional form	
Non-linear	1 (100)
DLNM usage	
No	1 (100)
Modeling framework^a^|Extreme events (n = 8)	
Self-established^a^	8 (100)
Statistical model	
(Quasi-)Poisson	8 (100)
GLM	1 (13)
GAM	1 (13)
Functional form	
Non-linear	6 (75)
Linear	1 (13)
Not mentioned	1 (13)
DLNM usage	
Yes	7 (88)
No	1 (13)

GBD, Global Burden of Disease; MCC, Multi-Country Multi-City; GWP, Gridded Population of the World; WHO, World Health Organization; AOD, aerosol optical depth; GLM, Generalized linear model; GAM, generalized additive model; MR-BRT, Meta-Regression—Bayesian, Regularized, Trimmed; CRA, Comparative risk assessment.

This table outlines data sources, modeling approaches, and analytical frameworks. Percentages are calculated relative to the total number of studies reporting each parameter.

a Study characteristics can be represented in multiple instances.

### The most frequent exposure–health relationships

**Fig. 3:**
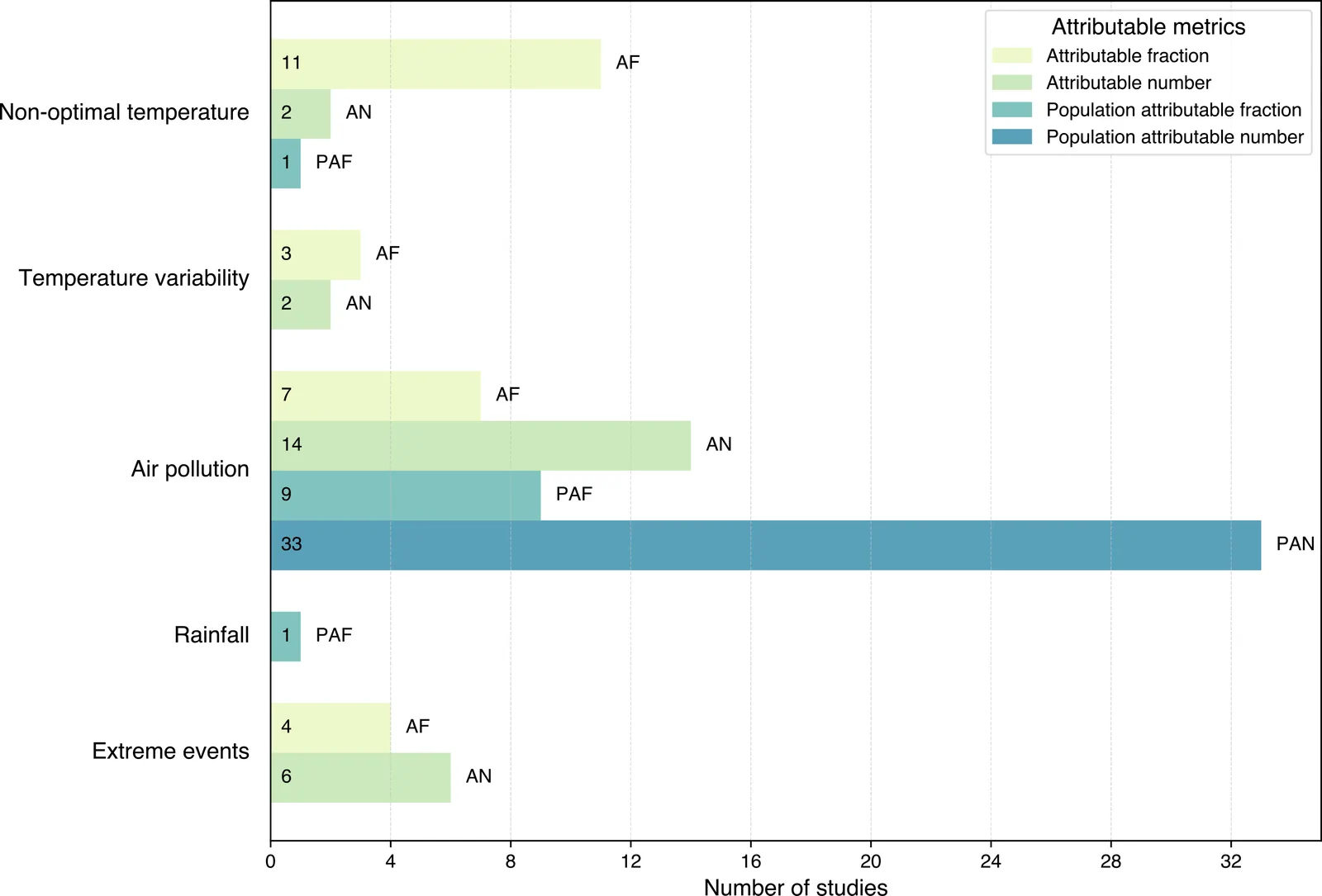
Distribution of attributable metrics, including attributable fraction (AF), attributable number (AN), population attributable fraction (PAF), population attributable number (PAN), used across climate-health studies in Southeast Asia (n = 77).

Studies commonly employed population attributable number (PAN), attributable fraction (AF), and population attributable fraction (PAF) as metrics (Fig. 3, Tables 1, 3, and 4). Methodological heterogeneity was pronounced, with 13 distinct comparison levels for PM_2_._5_, including WHO guidelines, national standards, and zero-exposure scenarios (Table 3).

**Table 3:** Summary of climate–health attribution studies in Southeast Asia, stratified by exposure type, health outcome, and attribution metric (n = 77).

Author; year	Location; time period	Exposure	Comparison	Attribution measure	Health outcome (ICD10)
Non-Optimal temperature					
Cao et al., 2021^34^	Thailand	Total: Heat + cold Heat: apparent temperature (temp) > MMT Cold: apparent temp < MMT	Minimum mortality temperature (MMT)	AF	Mortality All-cause
Cheng et al., 2020^35^	Vietnam	Heat: temp >95th	MMT	AF AN	Morbidity Dengue
Chua et al., 2022^36^	Philippines	Non-optimal temp: temp >95th + <5th	MMT	AF	Mortality Morbidity Enteric infections
Dang et al., 2018^37^	Vietnam	Heat (all): temp >75th Heat (mild): temp between 75th and 99th Heat (extreme): temp >99th	75th	AF	Mortality All-cause
Denpetkul et Phosri; 2021^23^	Thailand	Heat: temp > MMT Cold: temp < MMT	MMT	AF	Mortality All-cause Other
Gasparrini et al., 2015^38^	Thailand	Heat: temp > MMT Cold: temp < MMT	MMT	AF	Mortality All-cause
Nguyen et al., 2022^39^	Vietnam	Heat (all): temp >75th Heat (mild): temp between 75th–99th Heat (extreme): temp >99th	MMT	AF AN	Morbidity All-cause
Sritong-aon et al., 2025^40^	Thailand	Total: heat + cold Heat: temp > MMT Cold: temp < MMT	MMT	AF	Mortality All-cause
Thawonmas et al., 2025^41^	Thailand	Total: 0–100th Cool: 0–33rd Warm: 34–66th Hot: 67–100th	MMT	AF	Morbidity Suicides
Tobias et al., 2026^42^	Thailand	Heat > MMT Extreme heat >97.5th	MMT	AF	Mortality All-cause
Vicedo-Cabrera et al., 2021^11^	Philippines, Vietnam, Thailand	Heat: temp > MMT	MMT	AF	Mortality All-cause
Wen et al., 2024^43^	Thailand	Heat: temp > MMT Extreme heat: temp >97.5th Cold: temp < MMT Extreme cold: temp <2.5th	MMT	AF	Morbidity All-cause
Temperature variability					
Sritong-aon et al., 2025^44^	Malaysia, Philippines, Thailand	Diurnal temperature range (DTR)	Minimum Mortality DTR	AF AN	Mortality All-cause
Wu et al., 2022^45^	Thailand, Vietnam	Temperature variability (TV) stratified by quantiles	TV: SD of T_min_ and T_max_ for current day and 1 day before	AF	Mortality All-cause
Wen et al., 2024^46^	Philippines, Thailand, Vietnam	Temperature variability (interday, intraday)	Total deaths	AF AN	Mortality All-cause
Rainfall					
Tewari et al., 2024^47^	Singapore	Daily Rainfall, temperature, PM_10_	n/A	PAF	Morbidity Non-specific
Extreme events					
Huang et al., 2018^48^	Thailand	Heatwaves: 2–4 days of temp between: - mild: 90th–93rd, - medium: 94th–96th, - severe: 96th–99th	Non-heatwave days	AN	Mortality All-cause Other
Huang et al., 2023^49^	Myanmar, Philippines, Vietnam	Tropical cyclones (TC) (days with wind speed >17.5 m/s)	No TC days	AN	Mortality All-cause
Huang et al., 2024^50^	Thailand, Vietnam	Tropical cyclones (monthly TC days)	no TCs days	AF	Morbidity Infectious
Huang et al., 2025^51^	Thailand, Vietnam	Tropical cyclones (monthly TC days)	no TC days	AF AN	Mortality Circulatory
Phung et al., 2021^52^	Vietnam	River drought: water level <5th (77.5 cm)	Minimum hospitalization water level	AN	Morbidity All-cause Other
Tao et al., 2025^53^	Thailand	Heatwave: 3d over city specific threshold	Non-heatwave days	AF	Mortality All-cause
Yang et al., 2026^54^	Thailand, Vietnam	Flood days	Non-flood days	AN	Morbidity Infectious
Yang et al., 2025^55^	Thailand, Vietnam	Flood days	Non-flood days	AF AN	Morbidity All-cause Other
Air pollution					
Aik et al., 2020^56^	Singapore	PM_2.5_ (all and haze period), PM_10_ (all and haze period)	PM_2.5_: 10 μg/m^3^, PM_10_: 20 μg/m^3^	AN	Morbidity Conjunctivitis
Bui et al., 2023^57^	Vietnam	PM_2.5_	PM_2.5_: 5 μg/m^3^ (annual), 15 μg/m^3^ (daily)	PAN	Mortality Morbidity All-cause
Bui et al., 2021^58^	Vietnam	PM_10_	150 μg/m^3^	PAN	Mortality Morbidity All-cause Other
Bui et Nguyen; 2023^59^	Vietnam	PM_2.5_	50 μg/m^3^	PAN	Mortality Morbidity All-cause
Chen et al., 2021^60^	Philippines, Thailand, Vietnam	PM_2.5_ (wildfire-related)	0 μg/m^3^	PAF	Mortality Morbidity All-cause
Chen et al., 2024^61^	Philippines, Thailand, Vietnam	O_3_ (wildfire-related)	0 μg/m^3^	AF	Mortality All-cause Other
Chi et Oanh; 2021^62^	Thailand	PM_2.5_	2.4 μg/m^3^	PAF	Mortality All-cause Other
Chowdhury et al., 2021^63^	Indonesia	PM_2.5_	n/A	PAN	Mortality All-cause
Crippa et al., 2016^64^	Southeast Asia (all)	PM_2.5_ (wildfire-related)	0 μg/m^3^	PAN	Mortality All-cause
Fang et al., 2025^65^	Southeast Asia (all)	PM_2.5_	2.4 μg/m^3^	PAN	Mortality All-cause Other
Fink et al., 2026^66^	Southeast Asia (all)	PM_2.5_	7.5 μg/m^3^	PAF PAN	Mortality Lung cancer
Fold et al., 2020^67^	Thailand	PM_2.5_	Thai standard: 25 μg/m^3^ WHO guideline: 10 μg/m^3^	AN	Mortality All-cause Other
Hein et al., 2022^68^	Indonesia	PM_2.5_	Mean PM_2.5_ concentration during wet season	PAN	Mortality Morbidity All-cause Other
Hermayurisca et al., 2023^69^	Thailand	PM_2.5_	PM_2.5_: 5 μg/m^3^ (annual) 15 μg/m^3^ (daily)	PAF PAN	Mortality All-cause Other
Hu et al., 2026^70^	Southeast Asia (all)	O_3_	35 ppb	PAN	Mortality All-cause
Hystad et al., 2020^71^	Malaysia, Philippines	PM_2.5_	10 μg/m^3^	PAF	Mortality Morbidity Circulatory
Johnston et al., 2012^72^	Southeast Asia (all)	PM_2.5_ (wildfire-related)	Minimum regional wildfire-related PM_2.5_ concentration	AN	Mortality All-cause
Khempunjakul et al., 2025^73^	Thailand	PM_2.5_ + NO_2_	0 μg/m^3^	AF AN	Mortality All-cause
Lai et al., 2025^74^	Vietnam	PM_2.5_	WHO 15 μg/m^3^, Vietnam national standard: 50 μg/m^3^	PAN	Mortality Cardio-respiratory
Lee et al., 2018^75^	Southeast Asia (all)	PM_2.5_	5.8–8.8 μg/m^3^	PAN	Mortality All-cause
Lelieveld et al., 2013^76^	Southeast Asia (all)	PM_2.5_ + O_3_	0 μg/m^3^	PAN	Mortality Neoplasms Circulatory Respiratory
LinhNguyen et al., 2022^77^	Cambodia, Laos, Myanmar, Thailand, Vietnam	PM_2.5_ (all and biomass-burn-related)	0 μg/m^3^	PAN	Mortality All-cause Other
Lu et al., 2025^78^	Cambodia, Laos, Myanmar, Thailand, Vietnam	Fire-related PM_2.5_	0 μg/m^3^	PAN	Mortality Other
Marlier et al., 2012^79^	Southeast Asia (all)	PM_2.5_ (biomass burn-related), O_3_ (biomass burn-related)	0 μg/m^3^	PAN	Mortality Circulatory
Marlier et al., 2019^80^	Indonesia, Malaysia, Singapore	PM_2.5_ (wildfire-related)	0 μg/m^3^	PAN	Mortality All-cause
Mazeli et al., 2023^81^	Malaysia	PM_2.5_	PM_2.5_: 5 μg/m^3^	PAF PAN	Mortality All-cause
Mueller et al., 2021^82^	Thailand	PM_2.5_	2.4 μg/m^3^	AF AN	Mortality DALYs All-cause
Nguyen et al., 2024^83^	Vietnam	PM_2.5_, NO_2_	PM_2.5_: 5 μg/m^3^ NO_2_: 10 μg/m^3^	AF AN	Morbidity Respiratory
Nguyen et al., 2025^84^	Vietnam	PM_2.5_	Vietnam standard: 50 μg/m^3^	PAN	Mortality All-cause
Nhung et al., 2022^85^	Vietnam	PM_2.5_	Vietnam standard: 25 μg/m^3^ WHO: 10 μg/m^3^	AN	Mortality All-cause
Parntrasri et al., 2026^86^	Thailand	PM_2.5_	15 μg/m^3^	AN	Mortality Circulatory
Park et al., 2014^87^	Southeast Asia (all)	PM_2.5_ (wildfire-, non-fire related)	2.4 μg/m^3^	PAN	Mortality All-cause
Pinichka et al., 2015^88^	Thailand	O_3_	33.3–41.9 ppb	PAN	DALYs COPD
Pinichka et al., 2017^89^	Thailand	PM_10_, PM_2.5_, NO_2_	PM_10_: 10 μg/m^3^, PM_2.5_: 3 μg/m^3^, NO_2_: 0 μg/m^3^	PAF	Mortality All-cause Other
Punsompong et al., 2021^90^	Thailand	PM_2.5_	n/A	PAN	Mortality All-cause
Reddington et al., 2021^91^	Cambodia, Laos, Myanmar, Thailand, Vietnam	PM_2.5_	PM_2.5_: 2.4 μg/m^3^ O_3_: 26.7 ppb	PAN	Mortality DALYs All-cause
Sangkham et al., 2026^92^	Thailand	PM_2.5_	PM_2.5_: 3 μg/m^3^	AF	Mortality Cardio-respiratory Other
Shi et al., 2018^93^	Cambodia, Laos, Myanmar, Thailand, Vietnam	PM_2.5_	2.4–5.9 μg/m^3^	PAN	Mortality All-cause Other
Shi et al., 2018^94^	Cambodia, Laos, Myanmar, Thailand, Vietnam	PM_2.5_	2.4–5.9 μg/m^3^	PAN	Mortality All-cause
Sokharavuth et al., 2023^95^	Cambodia	PM_2.5_, NO_x_, SO_2_, NH_3_	5 μg/m^3^	PAN	Mortality All-cause
Sun et al., 2024^96^	Southeast Asia (all)	O_3_	40 ppb	PAN	Mortality All-cause
Syuhada et al., 2023^97^	Indonesia	PM_2.5_, O_3_	PM_2.5_: 2.4–5.9 μg/m^3^ (mean: 4.2 μg/m^3^), O_3_: 29.1–35.7 ppb (mean: 32.4 ppb)	AN	Morbidity Cardio-respiratory
Thongphunchung et al., 2022^98^	Thailand	PM_10_, PM_2.5_	NAAQS (25 μg/m^3^) and 25% of NAAQS (=NAAQS25)	AF AN	Mortality Morbidity I00-J99
Thongsame et al., 2025^99^	Thailand	PM_2.5_	7.5 μg/m^3^	PAN	Mortality Other
Uda et al., 2019^100^	Indonesia	PM_2.5_	10 μg/m^3^	AN	Mortality All-cause Other
Uttajug et al., 2022^101^	Thailand	Total PM_10_ (wildfire-related, including haze days)	Daily average PM_10_	PAF PAN	Morbidity Respiratory
Grosvenor et al., 2024^102^	Indonesia	Wildfire-related PM_2.5_	0 μg/m^3^	AN	Mortality All-cause
Xu et al., 2024^103^	Southeast Asia (all)	Fire-related PM_2.5_ + O_3_	0 μg/m^3^ PM_2.5_/O_3_	AN	Mortality All-cause
Xue et al., 2021^104^	Southeast Asia (all)	PM_2.5_ (wildfire-related)	n/A	AF AN	Mortality All-cause
Yin; 2022^105^	Cambodia, Laos, Malaysia, Myanmar, Thailand, Vietnam	PM_2.5_	2.4 μg/m^3^	PAN	Mortality All-cause
Yin; 2023^106^	Brunei, Indonesia, Malaysia, Singapore	PM_2.5_	2.4 μg/m^3^	PAN	Mortality All-cause
Zhang et al., 2022^107^	Indonesia, Philippines, Thailand	PM_2.5_	2.4–5.9 μg/m^3^	PAN	Mortality All-cause
Zhu et Shi; 2023^108^	Southeast Asia (all)	PM_2.5_	5 μg/m^3^	PAN	Mortality All-cause

AF, attributable fraction; AN, attributable number; PAF, population attributable fraction; PAN, population attributable number; mort, mortality; morb, morbidity; DALYs, disability adjusted life years lost; MMT, minimum mortality temperature; DTR, diurnal temperature range; Tmin, Minimum Temperature; Tmax, Maximum Temperature; NAAQS, National Ambient Air Quality Standards; NAAQS25, 25% of the National Ambient Air Quality Standards; PM_2.5_, Particulate Matter ≤2.5 μm; PM_10_, Particulate Matter ≤10 μm; O_3_, Ozone; NO_2_, Nitrogen Dioxide; SO_2_, Sulfur Dioxide; NOx, Nitrogen Oxides; NH_3_, Ammonia; CO, Carbon Monoxide; PSI, Pollutant Standards Index.

Only the metric of interest is reported; where available, AF/AN or PAF/PAN is shown, with no additional measures included. The label “other” denotes additional health outcomes reported in the Appendix (Table S2).

**Table 4:** Attribution metrics applied in Southeast Asian climate–health attribution studies (n = 77), with policy relevance as assessed by the study authors.

Metric	Definition	Usage	n	Typical use cases
Attributable Fraction (AF)	Percentage of health outcome attributable to exposure	34%	26	Non-optimal temperature, temperature variability, extreme events, air pollution
Attributable Number (AN)	Absolute number of cases attributable to exposure	31%	24	Non-optimal temperature, temperature variability, air pollution
Population Attributable Fraction (PAF)	Population-level percentage of disease burden due to exposure	12%	9	Air pollution, non-optimal temperature and rainfall
Population Attributable Number (PAN)	Estimated number of cases in the population due to exposure	43%	33	Air pollution

**Fig. 4:**
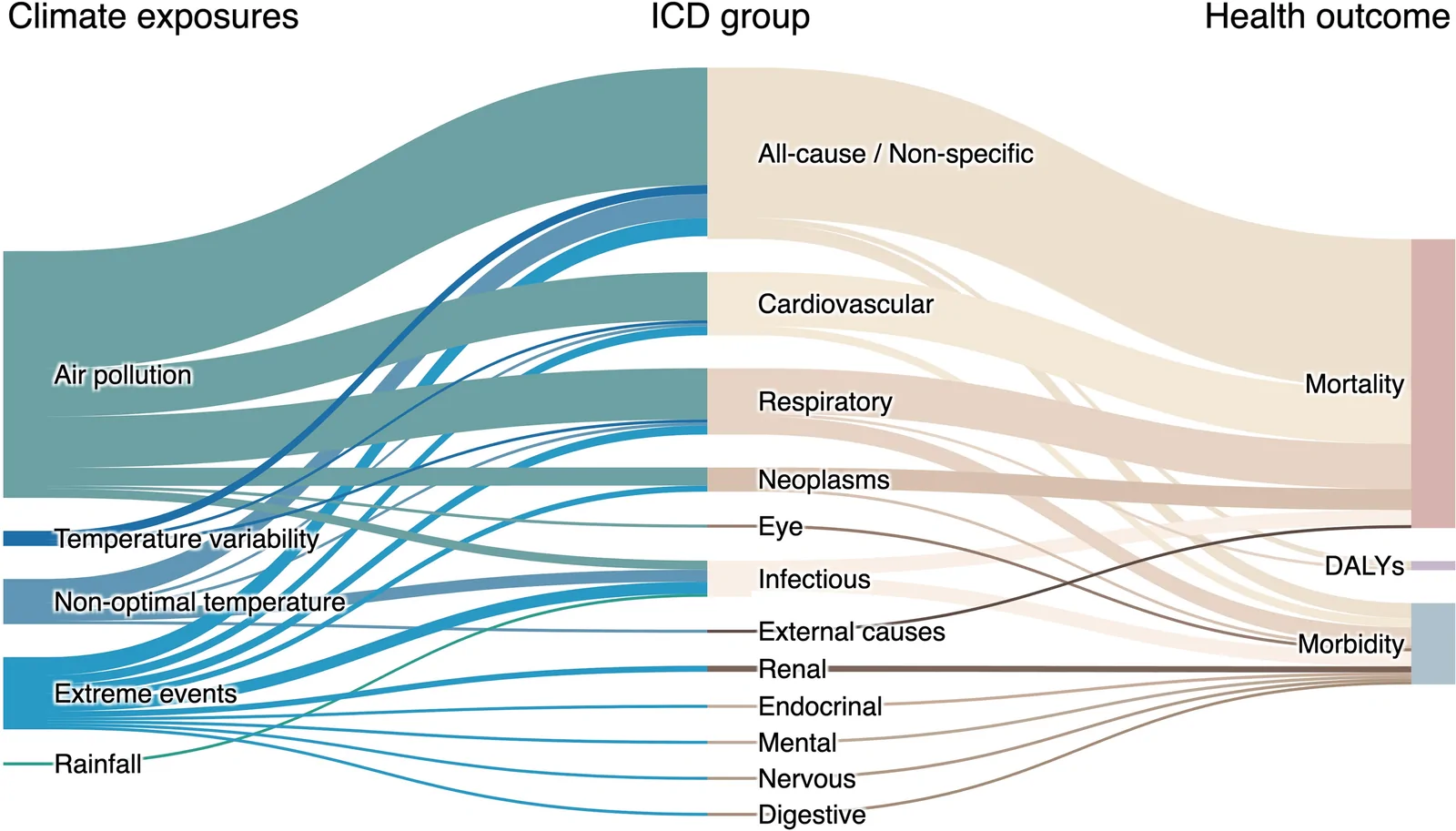
Sankey diagram illustrating the distribution of climate-health attribution studies by exposure type (left), ICD-10 disease group (center), and health outcome (right). Flow widths correspond to the number of studies linking specific exposures to disease categories and outcome types, highlighting dominant research pathways and thematic gaps.

As illustrated in Fig. 4, all-cause mortality represented the most frequently investigated outcome (n = 50; 65%), followed by cause-specific mortality across several ICD-10 categories: circulatory diseases (I00–I99; 23%, n = 18), respiratory diseases (J00–J99; 19%, n = 15), and cancer-related conditions (C00–D48; 9%, n = 7). Infectious diseases were examined less frequently, yet were assessed across both mortality (6%, n = 5) and morbidity outcomes (6%, n = 5). For mortality, external causes (suicides) were represented by only one study, indicating limited coverage beyond the dominant all-cause, cardiovascular, and respiratory outcomes. Morbidity estimates were also sparse for several ICD-10 categories, with neoplasms, eye diseases, mental disorders, digestive diseases, nervous system diseases, and endocrine diseases each represented by only one study, and renal outcomes by two studies. Several ICD-10 categories were absent altogether, highlighting substantial gaps in the current attribution evidence base. DALYs were employed as an outcome metric exclusively for all-cause and respiratory disease categories.

### Air pollution

Table 3 summarizes the exposure–health relationships reported across studies, while outcome numbers are provided in the Appendix (Table S4). AFs for all-cause mortality due to PM_2.5_ ranged from 0.7% to 31%, with the highest estimate reported in Thailand.^82^ PAN estimates were substantial: one regional study estimated 296,000 PM_2.5_-related deaths annually during El Niño years in Southeast Asia,^72^ while another modeled 1,147,259 deaths in this region in 2019, with further increases projected by 2050.^65^ Wildfire-related air pollution (PM_2.5_ + O_3_) accounted for 206,817 deaths in Southeast Asia, and wildfire-related O_3_ alone was linked to substantial mortality, particularly in Thailand (AF: 1.40%).^61^ DALY estimates indicated a burden exceeding 1 million years lost over 13 years across mainland Southeast Asia.^91^

Nine studies quantified morbidity, including over 117,000 hospital admissions and 162,000 emergency visits annually in Vietnam.^59^ PM_2.5_ was also linked to 635,000 asthma cases and 4390 respiratory hospitalizations in Indonesia.^68^

Several studies linked pollution exposure to high-impact events. For instance, haze-related PM levels during El Niño years drove surges in mortality and morbidity, with fire-related PM_2.5_ fractions reaching 11.8% in Indonesia and 10.2% in Myanmar.^104^

### Non-optimal temperature and variability

Temperature-related exposures were addressed in 21% of studies (n = 16), focusing on non-optimal temperatures, heat, cold, and temperature variability (TV). All studies developed their own exposure–response functions (ERFs), primarily using non-linear models (Table 2). Heat was consistently associated with increased all-cause mortality. In Bangkok, AFs reached 8.61%, with comparable burdens observed in other urban centers.^34^ Heat-related infectious disease burden included 18.5% of dengue cases during outbreaks in Vietnam.^35^ Non-optimal temperatures contributed 6–8% of outpatient and inpatient hospitalizations in Thailand.^43^ One study attributed up to 12% of suicide mortality to hot ambient temperatures in Thailand.^41^ Cold exposure, though less frequently analyzed, also showed notable impacts. In Thailand, cold accounted for 2.26% of infectious disease deaths, surpassing the burden attributable to heat.^23^

TV was increasingly studied, with all-cause mortality AFs rising alongside greater temperature fluctuations. For example, intraday TV yielded AFs of up to 1.17% in Vietnam, with corresponding PAN estimates exceeding 300 deaths annually.^46^

Climate projections suggest worsening impacts: Chua et al. estimated that enteric disease mortality linked to non-optimal temperatures could rise from 7.3% (2010–2019) to 33% by 2100 under high emissions (RCP8.5).^36^

### Extreme weather events and rainfall

Eight studies (10%) addressed extreme weather events, including heatwaves, tropical cyclones, floods, and droughts, of which 6 of 8 (88%) drew on data from Thailand. One study (1%) investigated respiratory infections linked to heavy rainfall.^47^

Heatwaves, typically defined as 2–4 consecutive days above the 90th percentile, were linked to substantial mortality. In Thailand, heatwaves accounted for 37,702 deaths over a decade, with higher mortality for neoplasms and infectious diseases than for cardiovascular outcomes,^48^ as well as an AF for deaths of 4.5% during heatwave periods.^53^

Studies focusing on flood-related hospitalizations reported negative (beneficial) associations across most investigated health outcomes, however one study reporting non-specific hospitalization AFs on flood days ranging from −0.27% (Vietnam) to 0.64% (Thailand), and respiratory disease AFs reaching −2.78%.^55^

Droughts and cyclones showed more variable impacts. In Vietnam, droughts were linked to 1134 hospitalizations, while tropical cyclones in Thailand contributed to 2447 hospitalizations for infectious diseases (AF: 0.38%).^50^^,^^52^

Rainfall extremes were strongly linked to morbidity in Singapore, where 29.1% of respiratory infection–related emergency visits were attributable to rainfall exceeding 20 mm/day.^47^

### Attribution metrics and methodological patterns

Overall, metric selection was heterogeneous, although most studies could be mapped onto established attributable-burden frameworks. Terminology varied substantially: estimates reported as excess deaths, premature mortality or similar, were therefore classified according to their mathematical equivalence to AF, AN, PAF, or PAN rather than by author-reported nomenclature. PAN was the most common metric (43%, n = 33), followed by AF (34%, n = 26) and AN (31%, n = 24), whereas PAF was less frequently applied (12%, n = 9). The frequent use of multiple metrics within single studies further illustrates the methodological flexibility of this literature, while underscoring the challenges for cross-study synthesis (Tables 1 and 4).

Air pollution studies were more likely to include both individual-level (AF/AN) and population-level (PAF/PAN) measures. For example, PAN values spanned from thousands to hundreds of thousands of attributable deaths per year, while AFs reached as high as 31% in high-exposure settings. Temperature studies primarily employed AF and AN; only one applied PAF, to infectious disease–related emergency visits. Metrics for rainfall and extreme events were rare and often specific to individual study designs (Fig. 3).

### ERF derivation and modeling approaches

**Fig. 5:**
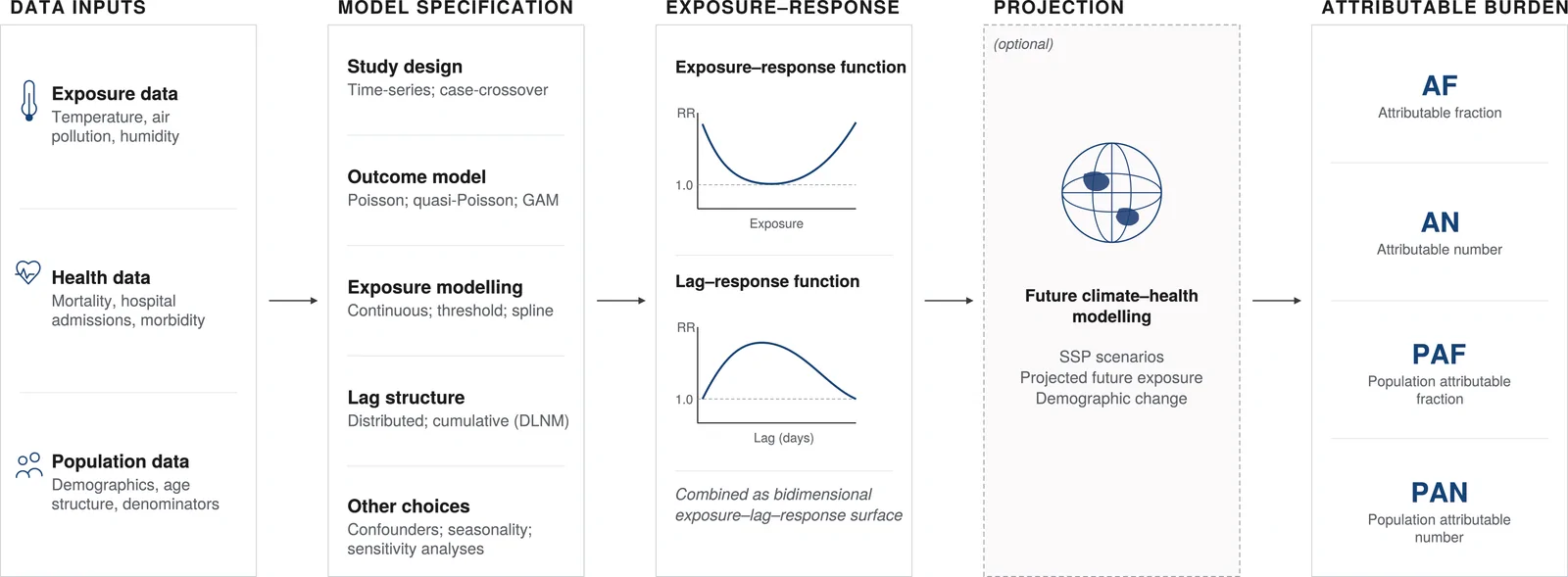
Conceptual workflow from data to attributable health metrics. This figure summarizes the analytical pathway from exposure, health, and population data through modeling choices, exposure–response function (ERF) estimation, optional projection under future climate-health scenarios, and derivation of attributable metrics, including attributable fraction (AF), attributable number (AN), population attributable fraction (PAF), and population attributable number (PAN).

ERFs formed the analytical basis for all attribution estimates; however, their derivation and underlying modeling choices varied substantially across studies, as shown in Fig. 5. Air-pollution studies predominantly relied on externally validated ERFs, most commonly the Integrated Exposure–Response model and the Global Exposure Mortality Model (GEMM) developed by Burnett et al.,^109^ alongside functions derived from the Meta-Regression—Bayesian, Regularized, Trimmed framework (MR-BRT),^28^ comparative risk assessment approaches, and other published sources, including functions proposed by Pope et al.^110^ Across the air-pollution studies, more than 36 distinct ERF sources were identified, highlighting substantial heterogeneity in risk-function selection. By contrast, all studies of temperature and extreme events derived ERFs within the primary study setting, typically using non-linear specifications to capture context-specific risk thresholds, such as the minimum mortality temperature (Table 2).

Among studies that self-derived ERFs, modeling strategies varied by exposure type. Quasi-Poisson regression was the most frequently used analytical framework for both air pollution and non-optimal temperature, although its use differed markedly between exposure groups: 64% of air-pollution studies and 92% of non-optimal temperature studies applied this approach, often within generalized linear model or generalized additive model frameworks (Table 2).

DLNMs were widely used to characterize delayed and non-linear exposure–response associations, but their application varied considerably by exposure type (Table 2). DLNM use was highest in studies of non-optimal temperature (92%, n = 12 of 13) and extreme events (88%, n = 7 of 8), and was also applied in two-thirds of temperature-variability studies (n = 2). In contrast, DLNMs were used infrequently in air-pollution studies with self-developed models (9%, n = 1 of 11), reflecting the predominance of burden estimates based on externally derived ERFs rather than study-specific exposure–lag modeling. Among studies applying DLNMs, lag specifications and degrees of freedom differed markedly across exposure types. Non-optimal temperature studies most often used daily lag windows extending up to 21 days, whereas air-pollution DLNMs generally used shorter acute lag periods, typically 0–2 or 0–10 days. Temperature-variability studies used heterogeneous lag specifications, including 0–21 and 0–10 days. Extreme-event studies incorporated the broadest lag structures, ranging from acute heatwave effects over 0–1 or 0–2 days to delayed health impacts of floods and tropical cyclones over several weeks or months. Reporting of DLNM degrees of freedom (df) was inconsistent; several studies reported spline bases or knot placement without specifying exposure or lag df. Detailed DLNM specifications are provided in Appendix (Table S3).

## Discussion

This review provides a systematic synthesis of climate-health attribution research in Southeast Asia, revealing a research landscape characterized by critical imbalances. Our findings confirm a growing body of evidence demonstrating substantial health burdens from climate-related exposures; air pollution was linked up to 31% of all-cause mortality, and heat was associated with up to 8.61% of deaths in some urban centers. However, this evidence base is critically skewed, with research dominated by a focus on air pollution and geographically concentrated in Thailand and Vietnam. These deep-seated geographic and methodological imbalances severely limit the ability to generalize findings and develop the effective, equitable, and region-wide adaptation strategies that are urgently needed.^111^

### Neglect of climate hazards and disease groups

A key finding from this review is the disproportionate focus on air pollution, which accounts for 70% of all included studies and predominantly examines mortality as an outcome (81%). This research concentration is largely driven by the high policy relevance of transboundary haze from biomass burning, which is particularly evident in Indonesia where it contributes to the highest AFs from peatland fires.^112^ The transboundary nature of this haze affects neighboring countries like Singapore, compounding the regional health burden. This situation is further exacerbated by climatic factors, as El Niño-induced droughts elevate PM_2.5_ concentrations and significantly increase mortality rates. The availability of globally standardized modeling frameworks, such as the Global Exposure Mortality Model (GEMM), also facilitates these analyses.^112^

Although episodic events such as certain climate exposures occur less frequently than more pervasive exposures, the predominant focus on a single hazard may be limiting, as it does not fully reflect the surrounding burden and may overlook other regionally important climate-related risks.^113^ Our review found that other climate-sensitive risk factors—such as heat, rainfall, and extreme events like heatwaves, floods and storms—were comparatively under-represented, while slow-onset phenomena like sea-level rise went unexamined.^91^^,^^114^^,^^115^ This imbalance should be interpreted in light of the episodic nature of extreme events such as heatwaves, floods, and storms, which occur less frequently than continuous exposures such as ambient temperature, rainfall, or air pollution. Their lower frequency reduces the number of observable exposure periods and can limit statistical power for attribution analyses, contributing to a smaller cumulative literature on these hazards. The under-representation of extreme events therefore reflects not only research-prioritization gaps but also an inherent event-frequency effect. Even allowing for this methodological constraint, the resulting evidence landscape remains uneven, with several regionally important climate hazards insufficiently quantified. This narrow exposure focus is mirrored by a similarly restricted outcome focus. Most studies concentrated on cardiovascular, respiratory, and all-cause outcomes, whereas attribution estimates for other disease groups, such as mental health, renal disease, digestive disease, nervous system disorders, endocrine disease, and external causes, remained sparse or entirely absent. This selective focus risks producing an incomplete picture of the overall disease burden, as unexamined health outcomes may nonetheless represent a considerable public health impact. Expanding attribution efforts to encompass these underexplored hazards and insufficiently examined disease groups is therefore warranted to establish an evidence base that more fully reflects the multifaceted nature of climate-related health risks across Southeast Asia.^98^

### Geographic disparities and the challenge to regional equity

A primary finding of this review, with profound implications for regional equity, is the severe geographic concentration of climate-health attribution research within Southeast Asia. Our analysis demonstrates that the existing evidence base is overwhelmingly dominated by studies situated in Thailand (n = 51, 66%) and Vietnam (n = 40, 52%), creating a deep but narrow evidence base rooted in the contexts of just two nations. In stark contrast, countries such as Brunei, Timor-Leste, Cambodia, and Laos remain markedly underrepresented or entirely absent from dedicated, in-country attribution studies, though they are occasionally included as part of larger regional models. This asymmetry, reflecting persistent disparities in research funding, technical capacity, and data infrastructure across the region, critically undermines efforts to build a coherent pan-regional understanding of climate-health risks. For underrepresented LMICs, support should target the structural conditions that determine whether climate-health evidence can be produced and translated into policy. Climate-health research funding should be channeled more directly to LMIC-based institutions and designed around local leadership, as recent evidence shows that only a very small share of health-and-climate research funding reaches primary research organizations in low- and lower-middle-income countries.^116^ Interoperable climate-health information systems are also needed to make routine health surveillance data more accessible and link them with meteorological, environmental, and climate information, consistent with the IPCC's emphasis on improved data collection, information-sharing, and modeling capacity in Asia.^117^ Support should further address gaps in ground-based meteorological monitoring outside major urban centers by expanding and maintaining local observation networks, as improved real-time observation data can strengthen assessment of hydro-meteorological hazards, exposure mapping, and early-warning capacity.^117^ In parallel, socio-political constraints on transboundary data-sharing should be addressed through regional cooperation, building on ASEAN experience in managing cross-border environmental risks, while recognizing that public-health data-sharing in Southeast Asia is constrained by differing standards, national rules, language barriers, capacity imbalances, and unstable financing arrangements.^118^ Together, these measures would help countries with currently limited evidence bases generate locally relevant climate-health knowledge, rather than relying on extrapolation from better-studied regional settings.

### Methodological fragmentation: a barrier to synthesis and policy application

Our findings contextualize a long-recognized gap in the global literature, affirming that methodological inconsistencies remain a key challenge in climate-health science, a trend highlighted in previous global assessments. Within Southeast Asia, however, our review reveals a more complex picture characterized by both promising signs of convergence and significant, persistent fragmentation.

As illustrated in Fig. 5, health attribution is analytically complex and highly dependent on the research question being addressed. The process begins with the definition of the exposure of interest and the selection of an appropriate attribution metric, such as AF or AN in temperature-related studies. It then requires careful specification of the input data, including whether exposure is derived from gridded products, weather-station observations, or modeled estimates, and whether the health endpoint is based on mortality, morbidity, or disease-specific outcomes. Further decisions are required at the modeling stage, ranging from the choice of statistical framework to the implementation of lag structures, non-linear exposure–response functions, and adjustment for confounding factors. For projection studies, additional layers of complexity arise from the decision to model future burden, the choice of projection year, and the climate, demographic, and socioeconomic scenarios applied. Our review identified a wide variety of data sources for health, meteorological, and population data, and this variability is most evident in the derivation of ERFs. While air pollution studies predominantly rely on externally validated ERFs derived from large, often global analyses, studies examining temperature and extreme events almost exclusively develop context-specific functions. At present, this approach represents best practice, given the absence of globally established ERFs that can serve as reliable proxies for temperature-related or extreme event burdens. The development of a globally applicable, high-resolution temperature ERF framework could substantially advance research in this area and enhance comparability across studies, provided it adequately captures local variability and context-specific risk patterns. However, this approach is even more challenging for extreme events, which are comparatively rare and highly heterogeneous, limiting the feasibility of robust, generalizable function estimation. This divergence reflects a fundamental trade-off between achieving local specificity and ensuring the cross-study comparability required for regional assessments. DLNM use supports the broader finding of methodological fragmentation: although widely applied in self-developed ERFs, especially for temperature and extreme events, specifications varied in lag windows, spline functions, knot placement, and reporting of degrees of freedom.

Ultimately, this mixed landscape—marked by partial convergence but persistent methodological fragmentation—remains a major barrier to quantitative synthesis. Greater harmonization across study design, from data transparency to exposure–response function derivation, is needed to improve comparability and strengthen the policy relevance of attribution research across Southeast Asia. The predominant use of AF and AN in temperature and extreme-event studies, and of PAF and PAN in air-pollution studies, suggests an emerging consensus that could be formalized as a field standard. Such standardization should extend to terminology: mathematically equivalent estimates are often reported under different labels, for example PAN-equivalent calculations described as excess deaths or premature mortality, which can lead to inconsistent classification of the same underlying attributable-burden approach.^49^^,^^75^ A parallel need exists for harmonized exposure definitions, particularly in heat-related research, where the boundary between non-optimal and extreme heat is inconsistently drawn; the 97.5th percentile of the local temperature distribution offers a defensible and reproducible cutoff. Counterfactual thresholds remain similarly heterogeneous: In total, 13 distinct thresholds were applied for PM_2.5_ alone, while temperature studies most commonly relied on the minimum mortality temperature (MMT). Convergence around established benchmarks—WHO Air Quality Guidelines or theoretical minimum-risk concentrations for PM_2.5_, and the MMT for non-optimal temperature exposures—would substantially improve cross-study comparability. For extreme weather events, however, the principal methodological challenge lies less in defining the counterfactual—typically the non-occurrence of the event—than in characterizing the exposure itself, including event duration, intensity, and spatial extent.

Standardization is equally warranted at the level of the modeling framework. The use of a 0–21-day lag window in 9 of 13 (69%) non-optimal-temperature DLNM studies indicates partial convergence around this specification. By contrast, other DLNM parameters—including exposure and lag degrees of freedom, spline basis, and knot placement—were inconsistently reported or too heterogeneous to define robust defaults. Across air-pollution studies, we identified more than 36 distinct sources of exposure–response functions, reflecting substantial methodological fragmentation. Pooled ERFs derived from large-scale meta-analyses, integrating evidence across regions and health outcomes, offer a robust foundation for harmonized burden estimation, and several are already widely applied, including GEMM and MR-BRT. For well-established exposure–outcome pairs, such as all-cause, cardiovascular, and respiratory mortality, these models can reasonably serve as default analytical tools. Authors should nevertheless remain attentive to the evolving literature, as updated formulations may better reflect contemporary evidence and regional specificity. Importantly, populations and health outcomes that are underrepresented in the underlying input data may be inadequately captured by such models, potentially leading to systematic over- or underestimation of the attributable burden.

### Strengths and limitations of the review

This study's primary strength is that it provides the first robust synthesis to map the climate–health attribution landscape in Southeast Asia. This was accomplished through a comprehensive search strategy across four major scientific databases, with a broad scope that included multiple climate exposures and health outcomes. However, the review is also subject to limitations. As a scoping review, its main purpose was to map existing evidence; the profound methodological heterogeneity identified across studies precluded any quantitative meta-analysis. Furthermore, our synthesis is constrained by limitations within the primary literature itself. We observed inconsistent terminology and definitions for exposures and outcomes, and a notable lack of sensitivity analyses in many studies, which complicates direct comparisons. The evidence base is also limited in its temporal scope, with rare use of future projections or climate counterfactuals, hindering a forward-looking assessment of health burdens. Our full-text retrieval criteria required that studies could be accessed through open access or the institutional access available to the review team. This restriction may still introduce selection bias by disproportionately excluding studies from lower-resourced institutions, older publications, or journals not covered by available institutional subscriptions, and may therefore understate the volume of evidence from under-represented nations such as Cambodia, Laos, Myanmar, and Timor-Leste. This bias acts in the same direction as the geographic imbalance reported in our findings and may lead us to overstate the degree of regional concentration. Finally, our search was restricted to studies published in English and may be subject to publication bias, whereby statistically significant findings are more likely to be published.

### Implications for policy and future research

Our findings highlight several urgent priorities to build a more robust and equitable evidence base for the region. Future studies should increasingly focus on at-risk populations, such as the elderly and indigenous groups, to better inform equity-focused interventions.

Despite the identified gaps, this review underscores the need for immediate and coordinated policy action to mitigate climate-related health risks in Southeast Asia. Existing ASEAN frameworks, such as the ASEAN Agreement on Transboundary Haze Pollution, illustrate the potential of regional cooperation in managing cross-border environmental risks, but similar coordination is needed for climate-health surveillance, data-sharing, and adaptation planning.^118^ This must be underpinned by coordinated regional action to enhance data sharing and resource mobilization across member states, and further reinforced through initiatives such as the Loss and Damage agenda of the Intergovernmental Panel on Climate Change (IPCC), through which economically stronger regions can provide support across financial, technological, and institutional dimensions.^119^

Health systems require enhanced capacity to manage the growing burden of climate-sensitive diseases, particularly respiratory and cardiovascular conditions.^120^ Key interventions should include the expansion of green spaces, especially tree canopies, and the implementation of early warning systems to protect vulnerable urban populations from heat exposure, alongside enhancing clinical readiness for climate-related health emergencies.^121^^,^^122^

This review confirms that Southeast Asia faces a significant and measurable health burden from climate-related exposures, particularly from air pollution and heat. However, the evidence required to mount an effective, equitable response remains critically incomplete and fragmented. The current research landscape is defined by deep geographic and methodological imbalances that limit the generalizability of findings and hinder the development of coherent, region-wide adaptation strategies. Addressing these gaps is essential. There is an urgent need for integrated, standardized research focused on underrepresented hazards and vulnerable nations to build the robust evidence base required to strengthen climate resilience across the region.

## Contributors

RS and SB conceptualized and designed the study. RS, MT, PB, SN, JC, TS, and SB developed the review framework. RS, MT, PB and JC did the literature search and data extraction. RS conducted the data analysis, data interpretation, and prepared the figures. SN and JC contributed to manuscript writing. TS provided secondary supervision. SB provided main supervision and contributed to study design and writing. All authors contributed to the writing and revision of the manuscript and approved the final version. RS attests that all listed authors meet authorship criteria and that no others meeting the criteria have been omitted. RS had full access to all data and takes responsibility for the decision to submit the manuscript.

## AI assistance disclosure

The authors used ChatGPT (OpenAI) to assist in improving the clarity and language of the manuscript. The authors reviewed and take full responsibility for the content of this publication.

## Declaration of interests

SB received grants from German research foundation. The rest authors declare no competing interests.
