# Supplementary material for: Attributing climate-related health burdens in Southeast Asia: a scoping review
Source: Lancet Reg Health West Pac. 2026 Aug 21;73:101952. doi: 10.1016/j.lanwpc.2026.101952 (PMC13524647; doi:10.1016/j.lanwpc.2026.101952)
Supplement: Supplementary file 1 [file mmc1.docx]

Supplementary Information

Attributing Climate-Related Health Burdens in Southeast Asia: A Systematic Evidence Synthesis

Robin Simion^1^, Min Thu^2^, Perla Boutros^1^, Syahrul Nellis^2^, Julia Feriato Corvetto^1^, Tin Tin Su^1,2^, Sandra Barteit^1^

^1^ Heidelberg Institute of Global Health (HIGH), Faculty of Medicine and University Hospital, Heidelberg University, Heidelberg, Germany

^2^ South East Asia Community Observatory (SEACO), Global Population Health, Jeffrey Cheah School of Medicine & Health Sciences, Monash University Malaysia, Bandar Sunway 47500, Malaysia, Malaysia­­­

Table of contents

[Figure S1 Prisma Checklist	4](#_Toc231737618)

[Search Strings	6](#_Toc231737619)

[Table S1 Included Studies	7](#_Toc231737620)

[Table S2 Main outcomes of included studies	32](#_Toc231737621)

[Table S3 DLNM extraction	53](#_Toc231737622)

Figure S1 Prisma Checklist

**Figure S1:** Preferred Reporting Items for Systematic reviews and Meta-Analyses extension for Scoping Reviews (PRISMA-ScR) Checklist

Search Strings

**Web of science** (1229 res.)

((ALL= (southeast asia OR brunei OR cambodia OR indonesia OR laos OR malaysia OR myanmar OR philippines OR singapore OR thailand OR timor-leste OR vietnam)) AND ALL=(climat* OR weather* OR temperature* OR heat* OR cold* OR drought* OR flood* OR rain* OR precipitation* OR storm* OR typhoon* OR air pollution* OR particulate matter*)) AND ALL=(health OR mortalit* OR death* OR hospitalization* OR morbidit* OR disease burden*) AND ALL=(attribut*)

**PubMed** (664 res.)

(asia, southeastern OR brunei OR cambodia OR indonesia OR laos OR malaysia OR myanmar OR philippines OR singapore OR thailand OR timor-leste OR vietnam) AND (climate OR weather OR temperature OR heat OR cold OR drought OR flood OR rainfall OR precipitation OR storm OR typhoon OR air pollution OR particulate matter) AND (health OR mortality OR death OR hospitalization OR morbidity OR disease burden) AND (attribution)

**Embase** (1229 res.)

(‚southeast asia’ OR brunei OR cambodia OR indonesia OR laos OR malaysia OR myanmar OR philippines OR singapore OR thailand OR ‚timor leste‘ OR vietnam) AND (climate OR weather OR temperature OR heat OR cold OR drought OR flooding OR rain OR precipitation OR hurricane OR ‚storm‘ OR ‚air pollution‘ OR ‚particulate matter‘)  AND (mortality OR death OR hospitalization OR ‚disease burden‘ OR health) AND attribut*

**Scopus** (2121 res.)

(southeast asia OR brunei OR cambodia OR indonesia OR laos OR malaysia OR myanmar OR philippines OR singapore OR thailand OR timor-leste OR vietnam) AND (climat* OR weather OR temperature OR heat OR cold OR drought OR flood OR rainfall OR precipitation OR typhoon OR storm OR air pollution OR particulate matter) AND (health OR mortality OR death OR hospitalization OR morbidity OR disease burden) AND (attribut*)

Last updated: April 2026

Table S1 Included Studies

| **Author year** | **Title** | **Country/ies in which the study conducted** | **Study design** | **Time period** | **Health data sources** | **Meteorological data sources** | **Population data sources** | **Weather exposure / Climate variable** | **Measured used for comparison** | **Statistical method used** |
| --- | --- | --- | --- | --- | --- | --- | --- | --- | --- | --- |
| Aik 2020 | The burden of acute conjunctivitis attributable to ambient particulate matter pollution in Singapore and its exacerbation during South-East Asian haze episodes | Singapore | Case /time series | 10 years | Weekly Infectious Disease Bulletin published by the Ministry of Health (Singapore) | Environmental Health Institute of the National Environment Agency, Singapore (NEA) and Meteorological Services Singapore (MSS) |  | PM2.5, PM10 | World Health Organization (WHO) air quality guideline (AQG) value | GAM regression model |
| Bui 2021 | Linking air quality, health, and economic effect models for use in air pollution epidemiology studies with uncertain factors | Vietnam | Case /time series | 1 month | none | WRF/CMAQ Model | 2019 Vietnam population and housing census | PM10 | National Ambient Air Quality Standards (150 μg/m3) | Chen 17, Bui 20 |
| Bui 2023 | Chronic and acute health effects of PM(2.5) exposure and the basis of pollution control targets | Vietnam | Case /time series | 1 year | Baseline from GBD 2019 | WRF/CMAQ |  | PM2.5 | WHO (15) /NAAQS (50) | IER (Burnett 14) |
| Bui et. Nguyen  2023 | Evaluation of the annual economic costs associated with PM2.5-based health damage-a case study in Ho Chi Minh City, Vietnam | Vietnam | Case /time series | 1 year | 2018 Health Statistical Yearbooks published by the Vietnamese Ministry of Health combined with: Institue for Health Metrics and Evaluation on GBD website. | WRF/CMAQ | Statistical Yearbook reports | PM2.5 | National Ambient Air Quality Standard (NAAQS) with a threshold value of 50 μg/m3 | Multiple ERF sources |
| Cao 2021 | The mortality effect of apparent temperature: A multi-city study in asia | Thailand; Other: China, Korea | Case /time series | 11 years | Thailand: Strategy and planning division | Thailand: Thailand Meteorological Department, the Ministry of Digital Economy and Society |  | Apparent temperature | Minimum mortality temperature (MMT) | Quasi-Poisson regression |
| Chen 2021 | Mortality risk attributable to wildfire-related PM(2·5) pollution: a global time series study in 749 locations | Philippines; Thailand; Vietnam | Case /time series | 17 years | MCC Collaborative Research Network (Mortality data) | GEOS-Chem |  | Wildfire related PM2.5 | 0 = No wildfire related PM2.5 in model | Quasi poisson regression |
| Chen 2024 | All-cause, cardiovascular, and respiratory mortality and wildfire-related ozone: a multicountry two-stage time series analysis | Philippines; Thailand; Vietnam | Case /time series | 17 years | MCC Network database | MCC Network database |  | Wildfire-related O3 | 0 O3 | Quasi poisson regression |
| Cheng 2020 | Heatwaves and dengue outbreaks in Hanoi, Vietnam: New evidence on early warning | Vietnam | Case /time series | 9 years | Hanoi Center for Preventive Medicine | [National Oceanic and Atmospheric Administration (NOAA)](https://www.noaa.gov/) |  | Temperature | 18°C (minimum dengue temperature) / no heatwave | GLM |
| Chi 2021 | Photochemical smog modeling of PM2.5 for assessment of associated health impacts in crowded urban area of Southeast Asia | Thailand | Case /time series | 1 year | GBD (2015) | WRF-CAMx Model | National statistics office | PM2.5 | 2.4ug/m3 | GEMM (Burnett 18) |
| Chowdhury 2022 | Global health burden of ambient PM2.5 and the contribution of anthropogenic black carbon and organic aerosols | Indonesia | Case /time series | 2 years | GBD | ECHAM/MESSy atmospheric chemistry (EMAC) general circulation model and compared with satelita image calculation | GBD 2019 population data | PM2.5 | GBD 2019 TMREL (2.4ug/m3) | GBD 2019 MR-BRT |
| Chua 2022 | Projecting Temperature-Attributable Mortality and Hospital Admissions due to Enteric Infections in the Philippines | Philippines | Case /time series | 4 years | Philippine Statistics Authority and the Philippine Health Insurance Corporation | ERA5-Land data set | Philippine Statistics Authority | Temperature (high and low) | Minimil mortality / morbidity temperature | Quasi-Poisson regression |
| Crippa 2016 | Population exposure to hazardous air quality due to the 2015 fires in Equatorial Asia | Brunei; Cambodia; Indonesia; Malaysia; Myanmar; Philippines; Singapore; Thailand; Timor-leste; Vietnam | Case /time series | 3 months | 2012 WHO Global Health Observatory | Modeled: WRF-Chem model + Satelites: (AOD) | 2013 LandScan High Resolution global Population Data | Air pollution (wilfire / haze) PM2.5 | No Haze = 0 PM2.5 | IER (Burnett 14) |
| Dang 2018 | Green Space and Deaths Attributable to the Urban Heat Island Effect in Ho Chi Minh City | Vietnam | Case /time series | 4 years | HCM City health department | 7 weather stations of the Hydro-Meteorological Data Center of Vietnam |  | Mild and extreme heat | Mild heat: 75th- 99th percentile Extreme heat: 99th- 100th percentile | Quasi-Poisson regression |
| Denpetkul 2021 | Daily ambient temperature and mortality in Thailand: Estimated effects, attributable risks, and effect modifications by greenness | Thailand | Case /time series | 8 years | Strategy and Planning Division under the Ministry of Public Health, Thailand | Thai Meteorological Department under the Ministry of Digital Economy and Society |  | Heat and Cold (> / < MMT) | MMT | Conditional Poisson regression |
| Fang 2025 | Effects of climate change on PM(2.5) formation chemistry, and the associated human health impacts and economic costs in Southeast Asia. | Brunei; Cambodia; Indonesia; Laos; Malaysia; Myanmar; Philippines; Singapore; Thailand; Timor-leste; Vietnam | Case /time series | 31 years | GBD (baseline & population) | WRF-CMAQ + CMIP6 (projection) | Gridded Population of the World (GPW) v4 | PM2.5 | 2.4 μg/m3 (theoretical minimum concentration) | GEMM (Burnett 18) |
| Fink 2026 | Global and regional cancer burden attributable to modifiable risk factors to inform prevention | Brunei; Cambodia; Indonesia; Laos; Malaysia; Myanmar; Philippines; Singapore; Thailand; Timor-leste; Vietnam | Case /time series | 10 ywars | GLOBOCAN 2022 (WHO cancer database) | N/A |  | PM2.5 | TMREL (7.5 µg/m³) | Hamra 14 |
| Fold 2020 | An assessment of annual mortality attributable to ambient PM2.5 in Bangkok, Thailand | Thailand | Case /time series | 1 year | Thailand Ministry of Public Health | 1 (2012) to 10 (2018) PM2.5 monitoring stations (Beta Ray attenuation method) | Self collected | PM2.5 | Thailand Standard: 25 µg/m3 (THAI) / WHO Guideline 10 µg/m3 (WHO) | Multiple ERF sources |
| Gasparrini 2015 | Mortality risk attributable to high and low ambient temperature: a multicountry observational study | Thailand | Case /time series | 19 years | Ministry of Public Health, Thailand | Meteorological Department, Ministry of Information and Communication Technology, Thailand |  | Temperature (high and low) | MMT | Quasi-Poisson regression |
| Grosvenor 2024 | Catastrophic impact of extreme 2019 Indonesian peatland fires on urban air quality and health | Indonesia | Case /time series | 17 years | World Bank death rate estimates for Indonesia | Sensor based on site | Gridded Population of the World (GPW) | Wildfire related PM2.5 | Wildfire-PM2.5 = 0 µg/m³ | Johnston 12 |
| Hein 2022 | The health impacts of Indonesian peatland fires | Indonesia | Case /time series | 5 years | Global Health Estimates (GHE) database by WHO based on official Indonesian national statistics as of year 2016 | PM2.5 concentrations are estimated using satellite-derived Aerosol  Optical Depth (AOD). | Gridded Population of the World (GPW) v4 | PM2.5 | Mean PM2.5 concentration in wet season | Vodonos 18 |
| Hermayurisca 2023 | Estimation of premature death attributed to short- and long-term PM2.5 exposure in Thailand | Thailand | Case /time series | 1 year | Thailand's Ministry of Public Health | Pollution Control Department Thailand |  | PM2.5 | 5 μg/m3; 15 μg/m3 | AirQ+ |
| Hu 2026 | Apportioning the ozone-related health impact to precursor emissions and formulating health-oriented ozone control strategies in Southeast Asia. | Brunei; Cambodia; Indonesia; Laos; Malaysia; Myanmar; Philippines; Singapore; Thailand; Timor-leste; Vietnam | Case /time series | 1 year | GBD | GEOS-Chem | GPWv4 / SEDAC | O3 | O3 = 35 ppb | Jerrett 09 |
| Huang 2018 | Mortality burden attributable to heatwaves in Thailand: A systematic assessment incorporating evidence-based lag structure | Thailand | Case /time series | 10 years | Ministry of Public Health, Thailand | Meteorological Department, Ministry of Digital Economy and Society, Thailand. | - | Heatwave | No heatwave days | Quasi-Poisson regression |
| Huang 2023 | Global short-term mortality risk and burden associated with tropical cyclones from 1980 to 2019: a multi-country time-series study | Myanmar; Philippines; Vietnam | Case /time series | 40 years | MCC Network | IBTrACS tropical cyclone data | World bank | Tropical cyclone (days with wind speed > 17.5 m/s) | No TC days | Conditional quasi-Poisson regression |
| Huang 2024 | Risks of infectious disease hospitalisations in the aftermath of tropical cyclones: a multi-country time-series study | Thailand; Vietnam | Case /time series | Thailand: 5 years; Vietnam: 13 years | Ministry of Health | Self modeled: International Best Track  Archive for Climate Stewardship (IBTrACS) | Gridded Population of the World (GPW) | Monthly number of tropical cyclones / windspeed | no TC | Quasi-Poisson regression |
| Huang 2025 | Hospitalization risk and burden for cause-specific cardiovascular diseases following tropical cyclones: A multicountry study. | Thailand; Vietnam | Case /time series | 20 years | National databases | Local wind profiles of tropical cyclones |  | Tropical cyclone | None - per TC day | Quasi-Poisson regression |
| Hystad 2020 | Associations of outdoor fine particulate air pollution and cardiovascular disease in 157 436 individuals from 21 high-income, middle-income, and low-income countries (PURE): a prospective cohort study | Malaysia; Philippines | Cohort study | 15.5 years | Primary data collection | Shaddick et al. 2018 — global PM2.5 estimates | PURE cohort, primarily collected | Ambient PM2.5 | 10µg/m³ | Cox proportional hazards frailty models |
| Johnston 2012 | Estimated Global Mortality Attributable to Smoke from Landscape Fires | Brunei; Cambodia; Indonesia; Laos; Malaysia; Myanmar; Philippines; Singapore; Thailand; Timor-leste; Vietnam | Case /time series | 10 years | Country-specific estimates for all-cause all-age mortal­ ity in the year 2002 were obtained from the  WHO Global Health Observatory (2011) | Satellite-derived observations of global fire activity (AOD) for PM2.5 |  | Landfire related PM2.5 | Minimum annual smoke-specific PM2.5 concentration (subregion level; not 0) | Comparative risk assessment |
| Khempunjakul 2025 | Mortality and economic burden of PM2.5 and NO2 in Thailand using satellite remote sensing and Random Forest algorithms | Thailand | Case /time series | 5 years | National statistical office Thailand | MODIS AOD |  | PM2.5 + NO2 | 0 µg/m³ | Chen et Hoek 20, Huang 21 |
| Lai 2025 | Health and economic impacts of PM2.5 exposure: A case study of pollution-related effects in Hanoi, Vietnam | Vietnam | Case /time series | 1 year | National Health Statistical Yearbook | WRF v3.8 + GFS global meteorological dataset; CMAQ model | Statistical Yearbook 2023 | PM2.5 | WHO AQG: 15 µg/m³ (24h average), Vietnam national standard (NTRAQ): 50 µg/m³ (24h average) | IER (Burnett 14) |
| Lee 2018 | Impacts of air pollutants from fire and non-fire emissions on the regional air quality in Southeast Asia | Brunei; Cambodia; Indonesia; Laos; Malaysia; Myanmar; Philippines; Singapore; Thailand; Timor-leste; Vietnam | Case /time series | 7 years | Baseline: WHO | WRF-Chem | National statistics of the respective countries | PM2.5 | 5.8-8.8 ug/m3 | Gu et Yim 16 |
| Lelieveld 2013 | Model calculated global, regional and megacity premature mortality due to air pollution | Brunei; Cambodia; Indonesia; Laos; Malaysia; Myanmar; Philippines; Singapore; Thailand; Timor-leste; Vietnam | Case /time series | 1 year | Baseline mortality rate and baseline YLL data from the WHO Statistical Information System on the country-level (WHO, 2012) | EMAC | CIESIN database | PM2.5 / O3 | 0 ug/m3 | PM2.5: Krewski 09; O3: Jerrett 09 |
| LinhNguyen 2022 | Public health and economic impact assessment of PM2.5 from open biomass burning over countries in mainland Southeast Asia during the smog episode | Cambodia; Laos; Myanmar; Thailand; Vietnam | Case /time series | 1 year | Baseline (Global Burdens of Disease) 2012 | PM2.5 by WRF-Community Multiscale Air Quality Modeling System (CMAQ) | Gridded Population of the World (GPW), version 4 (2010) | PM2.5 (BB related) | 0 PM2.5 | Multiple ERF sources |
| Lu 2025 | The contribution of fires to PM2.5 and population exposure in the Asia Pacific region | Other: Mainland / continental SEA | Case /time series | 7 years | GBD database for baseline (strope copd Ihd lung cancer) | ERA5, Modis MAIAC AOD, MERRA-2 AUD | LandScan (Oak Ridge National Laboratory) | Fire-related PM2.5 | 0 µg/m³ | IER (Burnett 14), Song 17 |
| Marlier 2013 | El Niño and health risks from landscape fire emissions in southeast Asia | Brunei; Cambodia; Indonesia; Laos; Malaysia; Myanmar; Philippines; Singapore; Thailand; Timor-leste; Vietnam | Case /time series | 10 years | Previous study | GEOS-Chem chemical transport model | United Nations Population Division and CIESIN’s Gridded Population of the World version 3 and Future Estimates, | PM2.5 from: peat, forest, agricultural waste burning, deforestation and degradation | No fire emissions | Pope 11, Bell 05 |
| Marlier 2019 | Fires, Smoke Exposure, and Public Health: An Integrative Framework to Maximize Health Beneﬁts From Peatland Restoration | Indonesia; Malaysia; Singapore | Case /time series | 5 years | GBD 2017 | GEOS-Chem | Gridded Population of the World (GPW) | Fire related PM2.5 | No fires | Vodonos 18, Mehta 11 |
| Mazeli 2023 | Cardiovascular, respiratory and all-cause (natural) health endpoint estimation using a spatial approach in Malaysia | Malaysia | Case /time series | 3x1 year | Institute for Public Health | Terra-MODIS (AOD) | Department of Statistics Malaysia website (DOSM, 2022) | PM2.5 | WHO | AirQ+ |
| Mueller 2021 | A health impact assessment of long-term exposure to particulate air pollution in Thailand | Thailand | Case /time series | 21 years | Thai Ministry of Public Health (MoPH) | Thai Pollution Control Department | Thai National Statistics Office | PM2.5 | 2.4 µg/m3 | GEMM (Burnett 18), Naghavi 17, Dimakakou 18 |
| Nguyen 2022 | The protective effect of green space on heat-related respiratory hospitalization among children under 5 years of age in Hanoi, Vietnam | Vietnam | Case /time series | 4 years | Three national hospitals in Hanoi (National Children’s  Hospital, National Hospital for Tropical Diseases, and Bach  Mai Hospital) | National Center for Hydro-Meteorological Forecasting + Weather Research and Forecasting (WRF) |  | Mild: Heat > 75th < 99th, Extreme: Heat > 99%, Total: > 75th-100th) | MMT | Quasi poisson regression |
| Nguyen 2024 | Effect of ambient air pollution on hospital admission for respiratory diseases in Hanoi children during 2007-2019 | Vietnam | Case /time series | 13 years | Hospital data base | Weather stations |  | PM2.5; NO2 | WHO: PM2.5: 5 ug/m3; NO2: 10 ug/m3 QCVN: WHO: 25 ug/m3; NO2: 40 ug/m3 | Logistic regression for linear, quasi poisson regreesion fo non linear |
| Nguyen 2025 | PM2.5 Pollution Assessment and Quantification of Short-Term Exposure Impacts on Public Health Using a Modeling Approach: A Case Study of Ca Mau Province, Vietnam | Vietnam | Case /time series | 4 months | National Health Statistical Yearbook | WRF/CMAQ | Vietnam Statistical Yearbook | PM2.5 | Vietnamese NAAQS 24h threshold: 50 µg/m³ | Bui 23 |
| Nhung 2022 | Mortality Burden due to Exposure to Outdoor Fine Particulate Matter in Hanoi, Vietnam: Health Impact Assessment | Vietnam | Case /time series | 1 year | Hanoi Center of Disease Control | Vietnam Environment Administration | Hanoi Center of Disease Control | PM2.5 | Vietnam Ambient National Standard of 25 μg/m3 and 10 μg/m3 | GEMM (Burnett 18) |
| Park 2014 | Future fire-PM2.5 mortality varies depending on climate and socioeconomic changes | Brunei; Cambodia; Indonesia; Laos; Malaysia; Myanmar; Philippines; Singapore; Thailand; Timor-leste; Vietnam | Case /time series | 10 years | GBD | GEOS-Chem |  | PM2.5 (fire vs. non-fire related) | GBD 2019 TMREL (2.4ug/m3) | GBD 2019 MR-BRT |
| Parntrasri 2026 | Weekly extremes of PM2.5-attributable cardiovascular mortality in Northeastern Thailand: Extreme-value return levels and 26-week forecasts | Thailand | Case /time series | 7 years | Ministry of Public Health Thailand | Thailand Pollution Control Department (PCD) | Department of Provincial Administration (DOPA) + National Statistical Office (NSO) | PM2.5 | 15 µg/m³ (WHO 24h guideline) | Orellano 20, Tabaei 23 |
| Phung 2021 | Hydropower dams, river drought and health effects: A detection and attribution study in the lower Mekong Delta Region | Vietnam | Case /time series | 37 years | Hospital records of two provincial (tertiary) hospitals for the period January 2011 – December 2014 | The daily river water level (RWL) was obtained from two hydrological monitoring stations (Can Tho and My Thuan). Daily meteorological data were obtained from the provincial hydrometeorological stations. |  | River drought < 5th precntile (77.5cm) | Threshhold RWL | GLM, poisson |
| Pinichka 2015 | Diseases Burden of Chronic Obstructive Pulmonary Disease (COPD) Attributable to Ground-Level Ozone in Thailand: Estimates Based on Surface Monitoring Measurements Data | Thailand | Case /time series | 1 year | BOD Thailand (Thai BOD) (BOD, 2009) | Thailand Pollution Control Departement (PCD) using monitoring stations in six regions for ground level Ozone and PM10 + GIS + Inverse distance weighting (IDW) | Gridded Population of the World (GPW) | O3 | 33.3–41.9 parts per billion | Comparative risk assessment |
| Pinichka 2017 | Burden of disease attributed to ambient air pollution in Thailand: A GIS-based approach | Thailand | Case /time series | 1 year | Verbal outopsy from BOD studies | We obtained air pollution data from the annual report on the state of air quality in Thailand, Pollution Control Department, Ministry ofNatural Resources and Environment | Gridded Population of the World (GPW), version 3 | PM10, PM2.5, (NO2) | 10 μgm-3 and 3 μgm-3 for PM10 and PM2.5, NO2 = 0 | Guo 14, Pope 02, Pope 2011, Faustini 2014 |
| Punsompong 2021 | Assessment of biomass-burning types and transport over Thailand and the associated health risks | Thailand | Case /time series | 1 year | Ministry of Public Health, Thailand | Pollution control department (PCD) has hourly resolution from 10 PM2.5 monitoring stations distributed in the four provinces. Thai AQI for 24h avarage, and Anual mean. | Gridded Population of the World (GPW) | PM2.5 | Table 1 | IER (Burnett 14) |
| Reddington 2021 | Air Pollution From Forest and Vegetation Fires in Southeast Asia Disproportionately Impacts the Poor | Cambodia; Laos; Myanmar; Thailand; Vietnam | Case /time series | 13 years | GBD 2017 | WRF-Chem | GPWv4 (CIESIN) | PM2.5 / Ozone | PM2.5 Minimum risk: 2.4 μg /m3.  O3: Minimum risk: 26.7 ppb. | GEMM (Burnett 18) + Turner 2016 |
| Sangkham 2026 | Estimated burden of disease and health effects attributable to fine particulate matter and ozone exposure in relation to COPD and cardiovascular disease in Upper Northern Thailand | Thailand | Case /time series | 9 years (long term), 4 years (short term) | Health Data Centre (HDC) dashboard | Thailand Pollution Control Department | Thailand population projections (Office of National Economic and Social Development Board) | PM2.5 (long term) + O3 (short term) | Long-term: nur PM2.5: 3 µg/m³ (background concentration) Short-term: Reference = 25 µg/m³ PM2.5 and 25 ppb O3 | Ostro 04, Bodor 22 |
| Shi 2018 | Underlying causes of PM2.5-induced premature mortality and potential health benefits of air pollution control in South and Southeast Asia from 1999 to 2014 | Cambodia; Laos; Myanmar; Thailand; Vietnam | Case /time series | 15 years | [GBD database](https://vizhub.healthdata.org/gbd-compare/) | Satellite-retrieved PM2.5 concentrations across SSEA (1999–2014) Donkelaaret al. (2016) GEIS Chem and AOD | [GPWv4 + Population Pyramids of the World](http://populationpyramid.net/) | PM2.5 | X0 denotes a theoretical minimum-risk concentration (range: 2.4–5.9 μg/m3), if higher they used other standards | IER (Burnett 14), Apte 15, Lelieveld 15 |
| Shi 2018 | Long-term trends and spatial patterns of PM2.5-induced premature mortality in South and Southeast Asia from 1999 to 2014 | Cambodia; Laos; Myanmar; Thailand; Vietnam | Case /time series | 16 years | Online GBD database | GEOS-Chem chemical transport model + AOD retrievals | GPWv4 + Population Pyramids of the World | PM2.5 | X0 represents the theoretical-minimum-risk concentration (range: 2.4–5.9 μg/m3) (Cohen et al., 2017) | IER (Burnett 14) |
| Sokharavuth 2023 | Air pollution mitigation assessment to inform Cambodia's first clean air plan | Cambodia | Case /time series | 6 years | GBD 2019 | Gridded dataset: IIASA GAINS ECLIPSE, combined with GEOS-Chem chemistry model | GPWv3 + UN Population Division | PM2.5, NOx, SO2, NH3 | WHO (anial mean of 5ug/m3)? | IER (Burnett 14) |
| Sritong-Aon 2025 | Temporal relationship between ambient temperature and non-accidental mortality in Thailand as evidence of inadequate adaptation to heat. | Thailand | Case /time series | 21 years | Strategy and Planning Division, Office of Permanent Secretary, Ministry of Public Health Thailand | Thai Meteorological Department (TMD) |  | Non-optimal temperature (heat and cold) | MMT | Quasi-Poisson regression |
| Sritong-Aon 2025 | Burden of mortality attributable to diurnal temperature range in Thailand: a nationwide case-crossover analysis from 2007 to 2021. | Thailand | Case-crossover | 15 years | Strategy and Planning Division, Office of Permanent Secretary, Ministry of Public Health Thailand | Thai Meteorological Department (TMD) |  | DTR | minimum mortality DTR (province specific) | Quasi-Poisson regression |
| Sun 2024 | An estimate of global cardiovascular mortality burden attributable to ambient ozone exposure reveals urban-rural environmental injustice | Brunei; Cambodia; Indonesia; Laos; Malaysia; Myanmar; Philippines; Singapore; Thailand; Timor-leste; Vietnam | Case /time series | 30 years | GBD 2019 Results portal — country-level annual age-stratified sex-standardized cardiovascular mortality rates; GBD baseline mortality | Fused O3 database (BayNND + CEML + M3-BME + TAP) | SEDAC GPW-v4 | Ambient O3 | TMREL: 40 ppb | NA |
| Syuhada 2023 | Impacts of Air Pollution on Health and Cost of Illness in Jakarta, Indonesia | Indonesia | Case /time series | 1 years | National Central Bureau of Statistics (BPS) Indonesia Health Care and Social Security Agency (BPJS) HAs: Healthcare and Social Security Agency | Environmental Agency of Jakarta Province | National Central Bureau of Statistics (BPS) | PM2.5, O3 | PM2.5: 2.4 µg/m3 to 5.9 µg/m3 (mean of 4.2 µg/m3); Ozone: 29.1 ppb to 35.7 ppb (mean of 32.4 ppb) | GBD 19 MR-BRT + multiple external sources |
| Tao 2025 | Quantifying localized heatwave impact on mortality: a multi-country modeling study in the Asia-Pacific region. | Thailand | Case /time series | up to 30 years | Ministry of Public Health Thailand | Thailand Meteorological Department |  | Heatwave: 3d over the Health-based temperature threshold (Th) (city specific temperature) | Health-based temperature threshold (Th) | Quasi poisson, GAM |
| Tewari 2024 | Associations Between Anthropogenic Factors, Meteorological Factors, and Cause-Specific Emergency Department Admissions | Singapore | Case /time series | 5 years | Ministry of Health, Singapore | Other: National Environment Agency Air pollution: Air Quality Open Data Platform. |  | PM2.5, Temp, Rainfall | 0 exposure | GAM, Poisson |
| Thawonmas 2025 | Ambient Temperature and Suicide Risk in Thailand: Evidence from Chiang Mai and Bangkok Provinces. | Thailand | Case-crossover | 20years | Ministry of Public Health Thailand | Thai Meteorological Department | National Statistical Office Thailand | Ambient temperature: cool: 0-33rd percentile, warm: 34-66th percentile, hot: 67-100th) | MMT (MinST (minimum suicide temperature )) | Conditional Poisson regression |
| Thongphunchung 2022 | Outpatient Department Visits and Mortality with Various Causes Attributable to Ambient Air Pollution in the Eastern Economic Corridor of Thailand | Thailand | Case-crossover | 7 years | National Health Security Office (NHSO) and Strategy and Planning Division of the Thailand Ministry of Public Health. | Fixed ambient air monitoring stations operated by the Thailand Pollution Control Department. | National Health Security Office (NHSO) | PM10, PM2.5, NO2, SO2, O3 | PM10: 1 µg/m3 increase  PM2.5: 1 µg/m3 increase NO2: 1 ppb increase SO2: 1 ppb increase O3: 1 ppb increase | Poisson model |
| Thongsame 2025 | Source Attribution and Health Burden of PM2.5 in Mainland Thailand | Thailand | Case /time series | 4 months | GBD 2019 (Murray et al. 2020) — baseline mortality rates (BMR) by disease and age group | WRF-Chem v4.2.2 | WorldPop 2019 | PM2.5 | GBD 2019 TMREL (2.4ug/m3) | GBD 2019 MR-BRT |
| Tobias 2026 | Variation in reporting of heatstroke mortality: evidence from a multi-country study | Malaysia; Philippines; Thailand | Case /time series | 23 years | National statistical offices | ERA5 (3 hottest months) | World Bank World Development Indicators Database | Heat > MMT, Extreme heat > 97.5th | MMT | Quasi-Poisson regression |
| Uda 2019 | Assessing the health impacts of peatland fires: a case study for Central Kalimantan, Indonesia | Indonesia | Case /time series | 5 years | Central Kalimantan Health Department | Hybrid Single-Particle Lagrangian Integrated Trajectory (HYSPLIT) model | Central Kalimantan Statistical Bureau | PM2.5 | 10 μg/m3 | Ostro 04 |
| Uttajug 2022 | Estimation of hospital visits for respiratory diseases attributable to PM10 from vegetation fire smoke and health impacts of regulatory intervention in Upper Northern Thailand | Thailand | Case /time series | 5 years | Ministry of Public Health of Thailand for each province | Pollution Control Department of Thailand | Gridded Population of the World, version 4 | PM10 | Fire-originated PM10 | Mueller 20 |
| Vicedo-Cabrera 2021 | The burden of heat-related mortality attributable to recent human-induced climate change | Philippines; Thailand; Vietnam | Case /time series | P: 5y, T: 10y, V: 5y | [MCC Collaborative Research Network database](http://mccstudy.lshtm.ac.uk/) | [MCC Collaborative Research Network database](http://mccstudy.lshtm.ac.uk/) |  | Heat (4 warmes months) | MMT, + (Counterfactual scenario without cc) | Quasi-Poisson regression |
| Wen 2024 | Association of cause-specific hospital admissions with high and low temperatures in Thailand: a nationwide time series study | Thailand | Case /time series | 6.5 years | Ministry of Public Health in Thailand | European Centre for Medium-Range Weather Forecasts Reanalysis v5 (ERA-5) |  | PM10 | MMT | GLM, Poisson |
| Wen 2024 | Comparison for the effects of different components of temperature variability on mortality: A multi-country time-series study | Philippines; Thailand; Vietnam | Case /time series | P: 14 years T: 10 years V: 5 years | MCC Collaborative Network | ERA5 dataset |  | TV | Minimum TV | GLM, poisson |
| Wu 2022 | Fluctuating temperature modifies heat-mortality association around the globe | Thailand; Vietnam | Case /time series | 47 years | MCC Collaborative Research Network database | MCC Collaborative Research Network database | MCC Collaborative Research Network database | Temperature variability stratified by quantiles | TV was calculated as the standard deviation (SD) of the average of Tmin and Tmax for the current day and 1 day before (Tmax-lag0, Tmax-lag1, Tmin-lag0, Tmin-lag1). | GLM, poisson |
| Xu 2024 | Global, regional, and national mortality burden attributable to air pollution from landscape fires: a health impact assessment study. | Brunei; Cambodia; Indonesia; Laos; Malaysia; Myanmar; Philippines; Singapore; Thailand; Timor-leste; Vietnam | Case /time series | 20 years | GBD 2019 — annual all-cause, cardiovascular, respiratory mortality | GEOS-Chem | GBD 2019 population data | Landscape fire-sourced PM2.5 + O3 | 0 µg/m³ PM2.5/O3 | Short term: quasi-Poisson, DLM Long term: Chen & Hoek 20, O3: Sun 22 |
| Xue 2021 | Associations between exposure to landscape fire smoke and child mortality in low-income and middle-income countries: a matched case-control study | Indonesia; Myanmar; Philippines | Case control study | 15 years | Demographic and Health Surveys | GEOS-Chem + MERRA-2 + GFED4.1s | Demographic and Health Surveys | PM2.5 | Fire-sourced concentrations of PM2.5 | Conditional logistic regression |
| Yang 2025 | Hospitalization risks associated with floods in a multi-country study. | Thailand; Vietnam | Case /time series | 20 years | Heath ministry of Thailand and provincial-/city-level hospitals and covered all hospitalizations in 26 provinces for Vietnam | Flood: Darnouth Flood Observatory (DFO) + MODIS | Landscan | Flood days | Non flood days | Quasi-Poisson regression |
| Yang 2026 | Risk, burden, and trend of infectious disease hospitalisations associated with floods: a multicountry, time-series study. | Thailand | Case /time series | 10 years | Local authorities Thailand (Ministry of Public Health) | ERA5 (0.25°), Dartmouth Flood Observatory (DFO) — global flood catalogue 2000–2019 | LandScan — annual population | Flood exposure (binary: flooded vs non-flooded community-week) | Non-flood week (reference = 0 flood exposure) | Quasi-Poisson regression |
| Yin 2022 | Decadal changes in premature mortality associated with exposure to outdoor PM(2.5) in mainland Southeast Asia and the impacts of biomass burning and anthropogenic emissions | Cambodia; Laos; Malaysia; Myanmar; Thailand; Vietnam | Case /time series | 30 years | GBD for baseline, HR modeled | PM2.5 from satellite-based dataset (V4.GL.03) of Atmospheric Composition Analysis Group (ACAG) (AOD) and from atmospheric reanalysis of the Modern-Era Retrospective Analysis for Research and Applications (MERRA) (AOD). | Population Division of the United Nations (UN) and Gridded Population of the World | PM2.5 | 2.4 μg/m3 (theoretical minimum concentration) | GEMM (Burnett 18) |
| Yin 2023 | Effect of biomass burning on premature mortality associated with long-term exposure to PM2.5 in Equatorial Asia | Brunei; Indonesia; Malaysia; Singapore | Case /time series | 30 years | GBD 2019 | V4.GL.03 (ACAG) PM2.5 (AOD) | The Gridded Population of the World (GPW) and the United Nations (UN) Population Division | PM2.5 | PM2.5 exposure from biomass burning and fossil fuel combustion | GEMM (Burnett 18) |
| Zhang 2022 | The dynamics of cardiovascular and respiratory deaths attributed to long-term PM2.5 exposures in global megacities | Indonesia; Philippines; Thailand | Case /time series | 20 years | Baseline GBD, RR from IER | The MODIS, MISR and SeaWiFS Aerosol Optical Depth (AOD) with Geographically Weighted Regression (GWR) dataset provides average annual estimates of ground-level PM2.5 concentrations | SEDAC,2018 | PM2.5 | 2.4 to 5.9 μg/m | IER (Burnett 14) |
| Zhu & Shi 2023 | Spatio-temporal variations of PM2.5 concentrations and related premature deaths in Asia, Africa, and Europe from 2000 to 2018 | Brunei; Cambodia; Indonesia; Laos; Malaysia; Myanmar; Philippines; Singapore; Thailand; Vietnam | Case /time series | 19 years | GB | GEOS-Chem | LandScan | PM2.5 | 5 μg/m3 | GBD 2019 MR-BRT |

# Table S2 Main outcomes of included studies

**Table S2:** Summary of climate–health attribution studies in Southeast Asia, stratified by exposure type, health outcome, and attribution metric (n = 73). Only the metric of interest is reported; where available, AF/AN or PAF/PAN are shown, with no additional measures included.

| Author; Year | Location;  Time period | Exposure;  Comparison | Main study outcomes | | | Attributional measure | | | | | |  |  |
| --- | --- | --- | --- | --- | --- | --- | --- | --- | --- | --- | --- | --- | --- |
| Non-Optimal Temperature | | | | | | **AF** | **AN** | **PAF** | **PAN** | **IRR** | **%Δ** |  |  |
| Cao et al.;  2021 ^1^ | Thailand;  11 years | Exposure:  Total: Heat + cold  Heat: apparent temperature (temp) > MMT Cold: apparent temp < MMT  Comparison: MMT | All-cause mortality:  Chiang Mai  Total: 2.84% (−3.89; 8.83)  Heat: 2.67% (−4.5; 9.09)  Cold: 0.17% (−0.40; 0.71) Bangkok  Total: 8.91% (−3.82; 19.78)  Heat: 8.61% (−3.74; 19.45)  Cold: 0.31% (−0.06; 0.67) | | | **x** |  |  |  |  |  |  |  |
|  |  |  |  |  |  |  |  |  |  |  | |  |  |
| Cheng et al.;  2020 ^2^ | Vietnam;  9 years | Exposure:  Heat: temp > 95^th^  Comparison: MMT | Dengue cases:  Large outbreaks: 1148.1  Small outbreaks: 70.6 | | |  | **x** |  |  |  |  |  |  |
|  |  |  |  |  |  |  | | | | | |  |  |
|  |  |  | Dengue cases:  Large outbreaks: 18.5%  Small outbreaks: 1.1%  *Large and small outbreaks as 90^th^ and 50^th^ percentile of weekly dengue distribution* | | | **x** |  |  |  |  |  |  |  |
|  |  |  |  |  |  |  | | | | | |  |  |
| Chua et al.;  2022 ^3^ | Philippines;  4 years | Exposure:  Non-optimal temp:  temp > 95^th^ + < 5^th^  Comparison: MMT | Enteric infection-specific mortality:  Present (2010-2019)  RCP 2.6: 6.9% (1.8; 10.0)  RCP 8.5: 7.3% (2.0; 10.7)  Projection (2090-2100)  RCP 2.6: 8.2% (-1.2; 15.9)  RCP 8.5: 33.0% (−1.4; 57.8) | | | **x** |  |  |  |  |  |  |  |
|  |  |  |  |  |  |  | | | | | |  |  |
|  |  |  | Enteric infection hospitalizations:  Present (2010-2019)  RCP 2.6: 10.7% (7.1; 13.6)  RCP 8.5: 10.8% (7.1; 13.5)  Projection (2090-2100)  RCP 2.6: 10.7% (6.3; 14.0)  RCP 8.5: 16.0% (−1.2; 30.3) | | | **x** |  |  |  |  |  |  |  |
|  |  |  |  |  |  |  | | | | | |  |  |
| Dang et al.;  2018 ^4^ | Vietnam;  4 years | Exposure:  Heat (all): temp > 75^th^  Heat (mild): temp between 75^th^-99^th^, Heat (extreme): temp > 99^th^  Comparison: 75^th^ | All-cause mortality:  Central area:   Heat (total): 1.42% (-)  Heat (mild): 1.12% (-)  Heat (extreme): 0.3% (-) Outer area:   Heat (total): 1% (-)  Heat (extreme): 0.26% (-)  Heat (mild): 0.74% (-)  Urban Heat Island effect: 0.42% (-) | | | **x** |  |  |  |  |  |  |  |
|  |  |  |  |  |  |  | | | | | |  |  |
| Denpetkul and Phosri; 2021 ^5^ | Thailand;  8 years | Exposure:  Heat: temp > MMT, Cold: temp < MMT  Comparison: MMT | All-cause mortality:  Heat: 2.45% (1.39; 4.46)  Cold: 1.28% (0.52; 2.01)  Infectious-disease mortality:  Heat: 0.66% (−0.17; 1.47)  Cold: 2.26% (0.01; 4.38)  Respiratory-disease mortality:  Heat: 0.50% (−0.05; 1.01)  Cold: 2.51% (0.15; 4.75) | | | **x** |  |  |  |  |  |  |  |
|  |  |  |  |  |  |  | | | | | |  |  |
| Gasparrini et al.;  2015 ^6^ | Thailand;  19 years | Exposure:  Heat: temp > MMT, Cold: temp < MMT  Comparison: MMT | All-cause mortality:  Heat: 0.76% (0.65; 0.86)  Cold: 2.61% (2.31; 2.88) | | | **x** |  |  |  |  |  |  |  |
|  |  |  |  |  |  |  | | | | | |  |  |
| Nguyen et al.;  2022 ^7^ | Vietnam;  4 years | Exposure:  Heat (all): temp > 75^th^, Heat (mild): temp between 75^th^-99^th^, Heat (extreme): temp > 99^th^  Comparison: MMT | Non-specific hospitalizations:  Heat (total): 58 (−194; 259)  Heat (mild): 18 (−195; 195)  Heat (extreme): 40 (−6; 69) | | |  | **x** |  |  |  |  |  |  |
|  |  |  |  |  |  |  | | | | | |  |  |
|  |  |  | Non-specific hospitalizations:  Heat (total): 0.52% (−1.73; 2.30)  Heat (mild): 0.17% (−1.72; 1.74)  Heat (extreme): 0.35% (−0.06; 0.61) | | | **x** |  |  |  |  |  |  |  |
|  |  |  |  |  |  |  | | | | | |  |  |
| Sritong-aon et al., 2025 ^8^ | Thailand;  21 years | Exposure:  Total: heat + cold,  Heat: temp > MMT, Cold: temp < MMT  Comparison: MMT | All-cause mortality:  Total: 3.94% (2.55; 5.29)  Cold AF: 2.52% (1.57; 3.43)  Heat AF: 1.43% (0.63; 2.22) | | | **x** |  |  |  |  |  |  |  |
|  |  |  |  |  |  |  |  |  |  |  |  |  |  |
| Thawonmas et al., 2025 ^9^ | Thailand;  20 years | Exposure:  Total: 0-100^th^  Cool: 0-33^rd^,  Warm: 34-66^th^,  Hot: 67-100^th^  Comparison: MMT | Suicide mortality:  Total AF: 24.61% (10.78; 34.68)  Cold AF: 4.55% (1.63; 7.20)  Warm AF: 8.35% (2.76; 12.49)  Hot AF: 12.05% (6.32; 16.10) | | | **x** |  |  |  |  |  |  |  |
|  |  |  |  |  |  |  |  |  |  |  |  |  |  |
| Tobias et al., 2026 ^10^ | Thailand;  23 years | Exposure:  Heat > MMT,  Extreme heat > 97.5^th^  Comparison: MMT | Heatstroke mortality:  Heat:  Philippines: 0.0%  Thailand: 2.0%  Malaysia: Heat: 0.2%  Extreme heat:  Philippines: 0.0%  Thailand: 3.7%  Malaysia: 0.5% | | | **x** |  |  |  |  |  |  |  |
|  |  |  |  |  |  |  |  |  |  |  |  |  |  |
| Vicedo-Cabrera et al.; 2021 ^11^ | Philippines, Vietnam;  5 years  Thailand;  5 -10 years | Exposure:  Heat: temp > MMT,  Climate change: temp with anthropogenic influence minus temp without anthropogenic influence  Comparison: MMT | All-cause mortality:  Heat  Philippines: 1.74% (-0.32; 5.07)  Thailand: 2.39% (0.02; 5.59)  Vietnam: 3.05% (0.37; 17.51) Climate change-related  Philippines: 1.06% (-0.05; 3.25)  Thailand: 1.27% (0.01; 2.67)  Vietnam: 1.48% (0.33; 5.13) | | | **x** |  |  |  |  |  |  |  |
|  |  |  |  |  |  |  | | | | | |  |  |
| Wen et al.; 2024 ^12^ | Thailand; 6.5 years | Exposure:  Heat: temp > MMT  Extreme heat: temp > 97.5^th^  Cold: temp < MMT  Extreme cold: temp < 2.5^th^  Comparison: MMT | Non-specific hospital admission:  Non-optimal temperature  Outpatient: 7.57% (6.47; 8.39)  Inpatient: 6.17% (4.88; 7.20)  All heat  Outpatient: 6.71% (5.80; 7.41)  Inpatient: 4.50% (3.62; 5.19)  Extreme heat  Outpatient: 1.44% (1.36; 1.49)  Inpatient: 0.96% (0.86; 1.02)  All cold  Outpatient: 0.86% (0.67; 0.98)  Inpatient: 1.67% (1.26; 2.00)  Extreme cold  Outpatient: 0.21% (0.14; 0.24)  Inpatient: 0.24% (0.16; 0.28) | | | **x** |  |  |  |  |  |  |  |
|  |  |  |  |  |  |  | | | | | |  |  |
| Temperature Variability | | | | | | **AF** | **AN** | **PAF** | **PAN** | **IRR** | **%Δ** |  |  |
| Sritong-aon et al.,  2025 ^13^ | Malaysia,  Philippines,  Thailand;  15 years | Exposure:  Diurnal temperature range | All-cause mortality:  AF  lag: 0-7d: 1.88% (0.69; 3.03)  lag: 0-14d: 2.39% (0.75; 3.99)  lag: 0-21d: 4.67% (−1.14; 9.87) | | | **x** |  |  |  |  |  |  |  |
|  |  |  |  |  |  |  |  |  |  |  |  |  |  |
|  |  |  | AN  lag: 0-7d: 104,807 (38,466; 168,918)  lag: 0-14d: 133,239 (41,811; 222,437)  lag: 0-21d: 260,345 (−63,553; 550,238) | | |  | **x** |  |  |  |  |  |  |
|  |  |  |  |  |  |  |  |  |  |  |  |  |  |
| Wu et al.;  2022 ^14^ | Thailand,  Vietnam;  47 years | Exposure: Temperature variability (TV) stratified by quantiles  Comparison:  TV: SD of T_min_ and T_max_ for current day and 1 day before | All-cause mortality:  Both countries:  Q1: 0.55% (-0.83; 1.91)  Q2: 1.16% (-1.68; 3.87)  Q3: 2.22% (-1.49; 5.62)  Q4: 3.56% (-0.87; 7.36) | | | **x** |  |  |  |  |  |  |  |
|  |  |  |  |  |  |  | | | | | |  |  |
| Wen et al.;  2024 ^15^ | Philippines;  14 years,  Thailand;  10 years,  Vietnam;  5 years | Exposure:  Temperature variability (interday, intraday)  Comparison: Total deaths | All-cause mortality:  Philippines AF AN Interday: 75 (62; 88) 0.10% Intraday: 582 (524; 640) 0.82%  Thailand Interday: 294 (243; 344) 0.16% Intraday: 2388 (2152; 2624) 1.13%  Vietnam:  Interday: 35 (29; 41) 0.13% Intraday: 312 (281; 342) 1.17%  Cardiovascular:  Philippines  Interday: 50 (43; 58) 0.19% Intraday: 496 (426; 565) 0.92%  Thailand  Interday: 100 (85; 116) 0.30% Intraday: 2388 (2152; 2624) 1.46%  Vietnam  Interday: 15 (13; 18) 0.26% Intraday: 79 (68; 91) 1.34%  Respiratory:  Philippines  Interday: 14 (9; 19) 0.13% Intraday: 90 (68; 113) 0.88%  Thailand  Interday: 47 (30; 63) 0.21% Intraday: 322 (242; 402) 1.43%  Vietnam  Interday: 4 (2; 5) 0.17% Intraday: 28 (21; 35) 1.25% | | | **x** | **x** |  |  |  |  |  |  |
|  |  |  |  |  |  |  | | | | | |  |  |
| Rainfall | | | | | | **AF** | **AN** | **PAF** | **PAN** | **IRR** | **%Δ** |  |  |
| Tewari et al.;  2024 ^16^ | Singapore;  5 years | Exposure: Temperature, daily Rainfall, PM10  Comparison: 0 exposure | Infectious diseases: Emergency department visits:  Temp peak: (29°C): 3.8% (2.1; 5.4)  Respiratory infections  Daily rainfall > 20mm/m^2^: 29.1% (22.2; 36.1)  PM10: 14.7% (9.3; 20.1) | | |  |  | **x** |  |  |  |  |  |
|  |  |  |  |  |  |  | | | | | |  |  |
| Extreme Events | | | | | | **AF** | **AN** | **PAF** | **PAN** | **IRR** | **%Δ** |  |  |
| Huang et al.;  2018 ^17^ | Thailand;  10 years | Exposure:  Heatwaves:  2-4 days of temp between:   - mild: 90^th^ - 93^rd^, - medium: 94^th^ - 96^th^, - severe: 96^th^ - 99^th^   Comparison: Non-heatwave days | Heatwave-related mortality:  All-cause Mild: 19686, medium: 12329, severe: 5687  Infectious diseases  Mild: 4566, medium: 3199, severe: 1345  Neoplasms  Mild: 6622, medium: 3977, severe: 1867  Cardiovascular diseases  Mild: 2556, medium: 2125, severe: 1081  Respiratory diseases  Mild: 2751, medium: 1569, severe: 781 | | |  | **x** |  |  |  |  |  |  |
|  |  |  |  |  |  |  | | | | | |  |  |
| Huang et al.;  2023 ^18^ | Myanmar,  Philippines,  Vietnam;  40 years | Exposure:  Tropical cyclones (TC)  (days with wind speed > 17.5 m/s)  Comparison: no TCs days | All-cause mortality  Per decade:  1889-1999: 24031 (13996; 36572)  2000-2019: 25507 (16588; 37518)  Total period: 24769(16052; 35733) | | |  | **x** |  |  |  |  |  |  |
|  |  |  |  |  |  |  |  |  |  |  |  |  |  |
| Huang et al.;  2024 ^19^ | Thailand;  5 years,  Vietnam;  13 years | Exposure:  Tropical cyclones  (monthly TC days)  Comparison: no TCs days | Infectious disease-related hospital admission:  Thailand: 2447 (-555; 4738)  Vietnam: -176 (-2828; 1748) | | | **x** |  |  |  |  |  |  |  |
|  |  |  |  |  |  |  | | | | | |  |  |
|  |  |  | Thailand: 0.38% (-0.09; 0.73)  Vietnam: -0.04% (-0.63; 0.39) | | |  | **x** |  |  |  |  |  |  |
|  |  |  |  |  |  |  | | | | | |  |  |
| Huang et al.,  2025 ^20^ | Thailand;  5 years,  Vietnam;  13 years | Exposure:  Tropical cyclones (monthly TC days)  Comparison: no TC days | Cardiovascular mortality:  Thailand: 1.07% (0.71; 1.32)  Vietnam: 1.41% (0.61; 1.73) | | | **x** |  |  |  |  |  |  |  |
|  |  |  |  |  |  |  |  |  |  |  |  |  |  |
|  |  |  | Thailand: 4,062 (2,696–4,998)  Vietnam: 6,854 (2,961–8,389) | | |  | **x** |  |  |  |  |  |  |
|  |  |  |  |  |  |  |  |  |  |  |  |  |  |
| Phung et al.;  2021 ^21^ | Vietnam;  20 years | Exposure:  River drought: water level < 5^th^ (77.5cm)  Comparison: Minimum hospitalization water level | River drought-related hospitalizations:  All-cause: 1134  Respiratory diseases: 115  Renal diseases: 158 | | |  | **x** |  |  |  |  |  |  |
|  |  |  |  |  |  |  | | | | | |  |  |
| Tao et al.,  2025 ^22^ | Thailand;  < 30 years | Exposure:  Heatwave: 3d over city specific threshold  Comparison: Non-heatwave days | All-cause mortality:  Thailand: 4.50% (3.33; 5.58) | | | **x** |  |  |  |  |  |  |  |
|  |  |  |  |  |  |  | | | | | |  |  |
| Yang et al.;  2026 ^23^ | Thailand,  10 years | Exposure:  Flood days  Comparison:  Non-flood days | **Total infectious diseases** hospitalizations:  Excess rate: −236.8 (per Million Person-years)  Attributable number (calculated): **−15,866** | | |  | **x** |  |  |  |  |  |  |
|  |  |  |  |  |  | Excess rate (out of AN) | | | | | |  |  |
|  |  |  |  |  |  |  |  |  |  |  |  |  |  |
| Yang et al.;  2025 ^24^ | Thailand,  Vietnam;  20 years | Exposure:  Flood days  Comparison:  Non-flood days | Hospitalizations:  **Non-specific:**   Thailand: −0.64% (−1.50; 0.18);  Vietnam: −0.27% (−0.65; 0.09)  **Cardiovascular:**  Thailand: −0.53% (−1.70; 0.58);  Vietnam: 0.09% (−0.35; 0.51)  **Respiratory:**  Thailand: −2.78% (−3.98; −1.65);  Vietnam: −0.47% (−0.86; −0.12)  I**nfectious diseases:**  Thailand: 0.84% (−0.32; 1.90);  Vietnam: −1.68% (−3.91; 0.23)  **Digestive:**  Thailand: −0.19% (−1.15; 0.69);  Vietnam: 0.29% (−0.22; 0.79)  **Mental:**  Thailand: 0.12% (−0.82; 1.02);  Vietnam: 0.57% (−1.06; 2.02)  **Diabetes:**  Thailand: 0.54% (−0.29; 1.32)  **Cancer:** Thailand: −0.13% (−1.06; 0.76)  **Nervous:** Thailand: 0.13% (−0.54; 0.75)  **Renal:** Thailand: 0.23% (−0.54; 0.97) | | | **x** |  |  |  |  |  |  |  |
|  |  |  |  |  |  |  |  |  |  |  |  |  |  |
|  |  |  | All-cause hospitalizations (annual):  Thailand: −16,056 (−37,566; 4,469)  Vietnam: −1,306 (−3,133; 443) | | |  | **x** |  |  |  |  |  |  |
|  |  |  |  |  |  |  |  |  |  |  |  |  |  |
| Air pollution | | | | | | **AF** | **AN** | **PAF** | **PAN** | **IRR** | **%Δ** |  |  |
| Aik et al.;  2020 ^25^ | Singapore;  10 years | Exposure:  PM_2.5_ (all and haze period), PM_10_ (all and haze period)  *Haze period = 1.7% of time*  Comparison:  PM_2.5_: 10 µg/m^3^,  PM_10_: 20 µg/m^3^ | Acute conjunctivitis cases:  Non-haze  PM_2.5_: 7911 (5458; 9940)  PM_10_: 7385 (5027; 9715)  Haze period  PM_2.5_: 1046 (710; 1366)  PM_10_: 1052 (731; 1358) | | |  | **x** |  |  |  |  |  |  |
|  |  |  |  |  |  |  | | | | | |  |  |
| Bui et al.;  2023 ^26^ | Vietnam;  1 year | Exposure: PM_2.5_  Comparison:  PM_2.5_: 5 µg/m^3^ (annual),  15 µg/m^3^ (daily) | All-cause mortality:  Total: 3109  Non-specific hospitalizations:  Total: 5213 | | |  |  |  | **x** |  |  |  |  |
|  |  |  |  |  |  |  | | | | | |  |  |
| Bui et al.;  2021 ^27^ | Vietnam;  1 month | Exposure: PM_10_  Comparison: 150 µg/m^3^ | Cause-specific mortality:  Infectious diseases: 0.008229 (0.01019; 0.025808) Respiratory diseases: 0.021717 (0.003825; 0.039375)  Hospitalizations:  Non-specific: 0.0026432 (0.0006204; 0.0043950) Infectious diseases: 0.0013366 (0.0001461; 0.0018588) Respiratory diseases: 0.0013066 (0.0004743; 0.0025362) | | |  |  |  | **x** |  |  |  |  |
|  |  |  |  |  |  |  | | | | | |  |  |
| Bui et Nguyen;  2023 ^28^ | Vietnam;  1 year | Exposure: PM_2.5_  Comparison: 50 µg/m^3^ | All-cause/Non-specific:  Mortality: 174 (16; 340)  Hospital admissions: 117632 (231850; 368926)  Emergency department visits: 162455 (-710465; 531153) | | |  |  |  | **x** |  |  |  |  |
|  |  |  |  |  |  |  | | | | | |  |  |
| Chen et al.;  2021 ^29^ | Philippines,  Thailand,  Vietnam;  17 years | Exposure:  PM_2.5_ (wildfire-related)  Comparison: 0 µg/m^3^ | All-cause mortality:  Philippines: 0.79% (0.62; 0.97)  Thailand: 2.32% (1.83; 2.80)  Vietnam: 0.99% (0.78; 1.21)  Infectious disease-related mortality:  Philippines: 0.80% (0.63; 0.98)  Thailand: 2.43% (1.92; 2.93)  Vietnam: 1.04% (0.81; 1.27)  Respiratory disease-related mortality:  Philippines: 0.78% (0.61; 0.95)  Thailand: 2.44% (1.92; 2.94)  Vietnam: 0.97% (0.76; 1.18) | | |  |  | **x** |  |  |  |  |  |
|  |  |  |  |  |  |  | | | | | |  |  |
| Chen et al.;  2024 ^30^ | Philippines,  Thailand,  Vietnam; 18 years | Exposure:  O_3_ (wildfire-related)  Comparison: 0 µg/m^3^ | All-cause mortality:  Philippines: 0.44% (0.24; 0.65)  Thailand 1.40% (0.76; 2.02)  Vietnam 0.74% (0.40; 1.08)  Cardiovascular mortality:  Philippines: 0.28% (–0.07; 0.63)  Thailand: 0.55% (–0.13; 1.22)  Vietnam: 0.38% (–0.09; 0.85)  Respiratory mortality:  Philippines: 0.49% (0.10; 0.88)  Thailand: 0.62% (0.13; 1.11)  Vietnam: 0.46% (0.10; 0.82) | | | **x** |  |  |  |  |  |  |  |
|  |  |  |  |  |  |  | | | | | |  |  |
| Chi and Oanh;  2021 ^31^ | Thailand;  1 year | Exposure: PM_2.5_  Comparison: 2.4µg/m^3^ | Mortality:  All-cause: 2.32% (1.83; 2.80)  Cardiovascular: 2.43% (1.92; 2.93)  Respiratory: 2.44% (1.92; 2.94)  All-cause:  Bangkok metropolitan domain: 3.3%  Bangkok city: 4.4%  Surrounding area: 2.1% | | |  |  | **x** |  |  |  |  |  |
|  |  |  |  |  |  |  | | | | | |  |  |
| Chowdhury et al.;  2021 ^32^ | Indonesia;  2 years | Exposure: PM_2.5_  Comparison: n/A | All-cause mortality:  113000 (78000; 167000)  *33% due to Biomass Burning* | | |  |  |  | **x** |  |  |  |  |
|  |  |  |  |  |  |  | | | | | |  |  |
| Crippa et al.;  2016 ^33^ | Brunei,  Cambodia, Indonesia, Malaysia, Myanmar, Philippines, Singapore, Thailand, Timor-Leste,  Vietnam; 3 months | Exposure:  PM_2.5_ (wildfire-related)  Comparison: 0 µg/m^3^ | All-cause mortality:  11880 | | |  |  |  | **x** |  |  |  |  |
|  |  |  |  |  |  |  | | | | | |  |  |
| ****Fang et al.; 2025 ^34^**** | Southeast Asia (all);  31 years | Exposure: PM_2.5_  Comparison: 2.4 µg/m³ | All-cause mortality:  SEA: 1,147,259 (982,811; 1,381,424) (year 2019)  Projection to 2090 (median):  SSP126: 82,345 (7.2%)  SSP245: -20,457 (-1.8%)  SSP585 109,608 (9.6%) | | |  |  |  | **x** |  |  |  |  |
|  |  |  |  |  |  |  |  |  |  |  |  |  |  |
| Fink et al.;  2026 ^35^ | Southeast Asia (all);  10 years | Exposure: PM_2.5_  Comparison: 7.5 µg/m³ | Lung, tracheal, bronchial cancer mortality: | | |  |  | **x** | **x** |  |  |  |  |
|  |  |  |  |  |  |  | | | | | |  |  |
|  |  |  | PAF:  1.0% | PAN:  13415 | | | | | | | | |  |
| Fold et al.;  2020 ^36^ | Thailand;  1 year | Exposure: PM_2.5_  Comparison:  Thai standard: 25 µg/m^3^  WHO guideline: 10 µg/m^3^ | All-cause mortality:  Thai standard: 1393 (593; 2691)  WHO: 3159 (893; 5248)  Neoplasm-related mortality:  Thai standard: 102 (45; 156)  WHO: 270 (125; 397)  Cardiopulmonary-related mortality:  Thai standard: 360 (284; 434)  WHO: 959 (769; 1140) | | |  | **x** |  |  |  |  |  |  |
|  |  |  |  |  |  |  | | | | | |  |  |
| Hein et al.;  2022 ^37^ | Indonesia;  5 years | Exposure: PM_2.5_  Comparison:  Mean PM_2.5_ concentration during wet season | All-cause mortality:  Adults: 33100  Infants: 2900  Respiratory disease hospitalizations:  4390  Asthma cases:  635000 | | |  |  |  | **x** |  |  |  |  |
|  |  |  |  |  |  |  | | | | | |  |  |
| Hermayurisca et al.; 2023 ^38^ | Thailand;  1 year | Exposure: PM_2.5_  Comparison:  PM_2.5_: 5 µg/m^3^ (annual)  15 µg/m^3^ (daily) | All-cause mortality: | | |  |  | **x** | **x** |  |  |  |  |
|  |  |  | PAF:  0.7% | | PAN:  1092 |  |  |  |  |  |  |  |  |
| Hu et al.;  2026 ^39^ | Southeast Asia (all);  1year | Exposure: O_3_  Comparison: 35 ppb | All-cause mortality Local: Singapore: 0, Malaysia: 727, Indonesia: 5038, Brunei: 0, Philippines: 404, Vietnam: 979, Cambodia: 11, Thailand: 1109, Laos: 0, Myanmar: 18, Timor-Leste: 0  Total SEA: 8286  Regional/transboundary sourced:  Singapore: 255, Malaysia: 730, Indonesia: 2175, Brunei: 0, Philippines: 34, Vietnam: 1435, Cambodia: 145, Thailand: 964, Laos: 28, Myanmar: 119, Timor-Leste: 0  Total SEA: 5885 | | |  |  |  | **x** |  |  |  |  |
|  |  |  |  |  |  |  |  |  |  |  |  |  |  |
| Hystad et al.;  2020 ^40^ | Malaysia, Philippines;  15.5 years | Exposure: PM_2.5_  Comparison: 10µg/m^3^ | Cardiovascular-related mortality:  8.3% (0.0; 15.2)    Cardiovascular-related cases:  3.9% (8.8; 18.6) | | |  |  | **x** |  |  |  |  |  |
|  |  |  |  |  |  |  | | | | | |  |  |
| Johnston et al.;  2012 ^41^ | Southeast Asia (all);  10 years | Exposure:  PM_2.5_ (wildfire-related)  Comparison:  Minimum regional wildfire related PM_2.5_ concentration | All-cause mortality:  Mean: 110000 / year  El Niño (dry): 296000 / year  La Niña (wet): 43000 / year | | |  | **x** |  |  |  |  |  |  |
|  |  |  |  |  |  |  | | | | | |  |  |
| Khempunjakul et al.;  2025 ^42^ | Thailand;  5 years | Exposure: PM_2.5_ + NO_2_  Comparison: 0 µg/m³ | All-cause mortality:  PM2.5: 0.79% (0.5; 1.09)  NO2: 0.6% (0.4; 0.79) | | | **x** |  |  |  |  |  |  |  |
|  |  |  |  |  |  |  |  |  |  |  |  |  |  |
|  |  |  | All-cause mortality (per year):  PM2.5: 20,487 (12,833; 28,079)  NO2: 15,394 (10,281; 20,487) | | |  | **x** |  |  |  |  |  |  |
|  |  |  |  |  |  |  |  |  |  |  |  |  |  |
| Lai et al.;  2025 ^43^ | Vietnam;  1 year | Exposure: PM_2.5_  Comparison:  WHO standard 15 µg/m³ (24h average),  Vietnam national standard: 50 µg/m³ (24h average) | Cardiorespiratory mortality:  WHO standard: 10297  Vietnam national standard: 2581  Cardiovascular mortality:  WHO standard: 4070  Vietnam national standard: 1020  Respiratory mortality:  WHO standard: 6227  Vietnam national standard: 1561 | | |  |  |  | **x** |  |  |  |  |
|  |  |  |  |  |  |  |  |  |  |  |  |  |  |
| Lee et al.;  2018 ^44^ | Southeast Asia (all);  7 years | Exposure: PM_2.5_  Comparison: 5.8-8.8 ug/m^3^ | All-cause mortality per year:  Jakarta (Indonesia): 910  Bangkok (Thailand): 1080  Hanoi (Vietnam): 620 | | |  |  |  | **x** |  |  |  |  |
|  |  |  |  |  |  |  |  |  |  |  |  |  |  |
| Lelieveld et al., 2013 ^45^ | Southeast Asia (all);  1 year | Exposure: PM_2.5_ + O_3_  Comparison: 0 ug/m^3^ | Cause-specific mortality:  PM_2.5_:  Lung cancer: 230000  CVD: 5790000  O_3_: Respiratory: 2330000 | | |  |  |  | **x** |  |  |  |  |
|  |  |  |  |  |  |  |  |  |  |  |  |  |  |
| LinhNguyen et al.; 2022 ^46^ | Cambodia,  Laos,  Myanmar, Thailand, Vietnam;  1 year | Exposure:  PM_2.5_ (all and biomass-burn-related)  Comparison: 0 µg/m^3^  *Impact of Biomass Burning on PM2.5: Cambodia: 54%, Laos: 70%, Myanmar: 69%, Thailand: 47%, Vietnam: 31%* | All-cause mortality:  Cambodia: 3625 (2895; 4345)  Laos: 11131 (9056; 13022)  Myanmar: 56514 (45392; 66894)  Thailand: 3629 (2890; 4330)  Vietnam: 3376 (2682; 4038)  Cardiovascular-related mortality:  Cambodia: 44 (20; 67)  Laos: 108 (51; 159)  Myanmar 629 (287; 942)  Thailand: 184 (83; 279)  Vietnam: 110 (49; 168)  Respiratory-related mortality:  Cambodia: 489 (258; 706)  Laos: 891 (496; 1227)  Myanmar 5001 (2707; 7066)  Thailand: 234 (124; 336)  Vietnam: 102 (54; 147) | | |  |  |  | **x** |  |  |  |  |
|  |  |  |  |  |  |  |  |  |  |  |  |  |  |
| Lu et al.;  2025 ^47^ | Mainland  Southeast Asia;  7 years | Exposure: Fire-related PM_2.5_  Comparison: 0 µg/m³ | Total SEA fire-related PM_2.5_ mortality:  58,000 (32,600; 82,600) | | |  |  |  | **x** |  |  |  |  |
|  |  |  |  |  |  |  |  |  |  |  |  |  |  |
| Marlier et al.;  2012 ^48^ | Southeast Asia (all);  10 years | Exposure:  PM_2.5_ (biomass burn-related),  O_3_ (biomass burn-related)  Comparison: 0 µg/m^3^ | Cardiovascular-related mortality:  El Niño 1997  PM_2.5_: 10800 (6800; 14300)  O_3_: 4100 (2300; 5900)  La Niña year 2000  PM_2.5_: 1600 (800; 2800)  O_3_: 1000 (600; 1400) | | |  |  |  | **x** |  |  |  |  |
|  |  |  |  |  |  |  | | | | | |  |  |
| Marlier et al.;  2019 ^49^ | Indonesia,  Malaysia,  Singapore;  5 years | Exposure:  PM_2.5_ (wildfire-related)  Comparison: 0 µg/m^3^ | All-cause mortality:  (2020-2029 projection)  Indonesia: 33000 (31000; 36000)  Malaysia: 2400 (2200; 2600)  Singapore: 360 (340; 380) | | |  |  |  | **x** |  |  |  |  |
|  |  |  |  |  |  |  | | | | | |  |  |
| Mazeli et al.;  2023 ^50^ | Malaysia;  3x1 year | Exposure: PM_2.5_  Comparison:  PM_2.5_: 5 µg/m^3^ | All-cause mortality:  PAN PAF  2000: 5893 6.96% (4.60; 9.12)  2008: 5091 4.7% (3.09; 6.18)  2013: 9781 8.08% (5.34; 10.56) | | |  |  | **x** | **x** |  |  |  |  |
|  |  |  |  |  |  |  | | | | | |  |  |
| Mueller et al.;  2021 ^51^ | Thailand;  21 years | Exposure: PM_2.5_  Comparison: 2.4 µg/m^3^ | All-cause mortality:  31% (21; 40) | | | **x** |  |  |  |  |  |  |  |
|  |  |  |  |  |  |  | | | | | |  |  |
|  |  |  | All-cause mortality:  50019 (42189; 57849)  All-cause DALYs:  508918 (438345; 579492) | | |  | **x** |  |  |  |  |  |  |
|  |  |  |  |  |  |  | | | | | |  |  |
| Nguyen et al.;  2024 ^52^ | Vietnam;  13 years | Exposure: PM_2.5_, NO_2_  Comparison:  PM_2.5_: 5 µg/m^3^  NO_2_: 10 µg/m^3^ | Hospital admissions: Respiratory  4.3% (3.5; 5.2) | | | **x** |  |  |  |  |  |  |  |
|  |  |  |  |  |  |  | | | | | |  |  |
|  |  |  | 1619 (1299; 1935) | | |  | **x** |  |  |  |  |  |  |
|  |  |  |  |  |  |  | | | | | |  |  |
| Nguyen et al.;  2025 ^53^ | Vietnam;  4 months | Exposure: PM_2.5_  Comparison:  Vietnam standard: 50 µg/m^3^ | All-cause mortality:  222 (−10; 342) | | |  |  |  | **x** |  |  |  |  |
|  |  |  |  |  |  |  |  |  |  |  |  |  |  |
| Nhung et al.;  2022 ^54^ | Vietnam;  1 year | Exposure: PM_2.5_  Comparison:  Vietnam standard: 25 µg/m^3^  WHO: 10 µg/m^3^ | All-cause mortality:  Vietnam standard: 2696 (2225; 3158)  WHO: 4760 (3958; 5534) | | |  | **x** |  |  |  |  |  |  |
|  |  |  |  |  |  |  | | | | | |  |  |
| Park et al.;  2014 ^55^ | Southeast Asia (all);  10 years | Exposure: PM_2.5_ (wildfire-, non-fire related)  Comparison: 2.4ug/m^3^ | Total all-cause mortality:  Baseline: 2010s  Brunei: 43 (28; 61) - 45.6% fire-related  Cambodia: 107 (88; 123) - 3.0% fire-related  Indonesia: 33752 (26533; 40360) - 32.5% fire-related  Laos: 123 (103; 139) - 1.9% fire-related  Malaysia: 5888 (4204; 7537) - 51.5% fire-related  Myanmar: 1006 (850; 1140) - 1.9% fire-related  Philippines: 1089 (874; 1285) - 10.1% fire-related  Singapore: 0 (0; 0) - 0.0% fire-related  Thailand: 2654 (2040; 3214) - 2.7% fire-related  Timor-Leste: 6 (5; 7) - 6.3% fire-related  Vietnam: 2362 (1925; 2742) - 2.6% fire-related  Projection: 2050s  Brunei: 25–52  Cambodia: 33–104  Indonesia: 28284–58895  Laos: 57–212  Malaysia: 5121–10597  Myanmar: 340–1143  Philippines: 897–2156  Singapore: 0  Thailand: 939–2883  Timor-Leste: 6–19  Vietnam: 1007–2836  Projection: 2090s  Brunei: 22–74  Cambodia: 20–253  Indonesia: 19814–68212  Laos: 42–479  Malaysia: 4588–15337  Myanmar: 166–1444  Philippines: 979–4893  Singapore: 0  Thailand: 545–3832  Timor-Leste: 3–55  Vietnam: 641–4073 | | |  |  |  | **x** |  |  |  |  |
|  |  |  |  |  |  |  |  |  |  |  |  |  |  |
| ****Parntrasri et al.; 2026 ^56^**** | Thailand;  7 years | Exposure: PM_2.5_  Comparison: 15 µg/m³ | Mortality:  Cardiovascular: 435,  Ischemic Heart Disease: 297 | | |  | **x** |  |  |  |  |  |  |
|  |  |  |  |  |  |  | | | | | |  |  |
| Pinichka et al.;  2015 ^57^ | Thailand;  1 year | Exposure: O_3_  Comparison: 33.3-41.9 ppb | COPD-related DALYs:  61577 | | |  |  |  | **x** |  |  |  |  |
|  |  |  |  |  |  |  | | | | | |  |  |
| Pinichka et al.;  2017 ^58^ | Thailand;  1 year | Exposure:  PM_10_, PM_2.5_, NO_2_  Comparison:  PM_10_: 10 µg/m^3^,  PM_2.5_: 3 µg/m^3^,  NO_2_: 0 µg/m^3^ | All-cause mortality:  PM_10_: 1.7%, PM_2.5_: 7.6%, NO_2_: 1.0%  Cause-specific mortality:  Lung cancer:  PM_2.5_: 16.9%, Cardiovascular diseases:  PM_10_: 3.8%, PM_2.5_: 14.6%, Respiratory diseases:  PM_10_: 1.7%, NO_2_: 2.5% | | |  |  | **x** |  |  |  |  |  |
|  |  |  |  |  |  |  | | | | | |  |  |
| Punsompong et al.; 2021 ^59^ | Thailand;  1 year | Exposure: PM_2.5_  Comparison: n/A | All-cause mortality:  18003 | | |  |  |  | **x** |  |  |  |  |
|  |  |  |  |  |  |  | | | | | |  |  |
| Reddington et al.; 2021 ^60^ | Cambodia, Laos, Myanmar, Thailand, Vietnam;  13 years | Exposure: PM_2.5_  Comparison:  PM_2.5_: 2.4 µg/m^3^  O_3_: 26.7 ppb | All-cause mortality:  PM_2.5_: 27500 (24700; 30400)  All-cause DALYs:  PM_2.5_: 1047500 (867500; 1247300) | | |  |  |  | **x** |  |  |  |  |
|  |  |  |  |  |  |  | | | | | |  |  |
| Sangkham et al.; 2026 ^61^ | Thailand;  9 years (long term), 4 years (short term) | Exposure:  Long-term: PM_2.5_  Comparison:  Long-term:  PM_2.5_: 3 µg/m³ | Lung cancer mortality:  Long term: 35.56% (34.12; 37.00)  Cardiopulmonary mortality:  Long term: 25.49% (24.40; 26.57) | | | **x** |  |  |  |  |  |  |  |
|  |  |  |  |  |  |  |  |  |  |  |  |  |  |
| Shi et al.;  2018 ^62^ | Cambodia, Laos, Myanmar,  Thailand,  Vietnam;  16 years | Exposure: PM_2.5_  Comparison: 2.4 to 5.9 µg/m^3^ | All-cause mortality:  Vietnam: 48900, Myanmar: 45700,  Thailand: 29500, Cambodia: 9000,  Laos: 5500  Cardiovascular disease mortality:  Vietnam: 3100, Myanmar: 1600, Thailand: 2700, Cambodia: 200, Laos: 100 | | |  |  |  | **x** |  |  |  |  |
|  |  |  |  |  |  |  | | | | | |  |  |
| Shi et al.;  2018 ^63^ | Cambodia, Laos, Myanmar,  Thailand,  Vietnam;  15 years | Exposure: PM_2.5_  Comparison: 2.4 to 5.9 µg/m^3^ | All-cause mortality:  Vietnam: 1100, Myanmar: 22400,  Thailand: 15500, Cambodia: 3700, Laos: 2700 | | |  |  |  | **x** |  |  |  |  |
|  |  |  |  |  |  |  | | | | | |  |  |
| Sokharavuth et al.; 2023 ^64^ | Cambodia;  6 years | Exposure:  PM_2.5_, NO_x_, SO_2_, NH_3_  Comparison: 5 µg/m^3^ | All-cause mortality:  2015: 3100 (1850; 4200) 2030: 5700 (3400; 7700) | | |  |  |  | **x** |  |  |  |  |
|  |  |  |  |  |  |  | | | | | |  |  |
| Sun et al.;  2024 ^65^ | Southeast Asia (all);  30 years | Exposure: O_3_  Comparison: 40 ppb | All-cause mortality:  Urban: 86000 (43000; 132000)  Rural: 198000 (100000; 303000) | | |  |  |  | **x** |  |  |  |  |
|  |  |  |  |  |  |  |  |  |  |  |  |  |  |
| Syuhada et al.; 2023 ^66^ | Indonesia;  1 year | Exposure: PM_2.5_, O_3_  Comparison:  PM_2.5_: 2.4 to 5.9 µg/m^3^ (mean: 4.2 µg/m^3^),  O_3_: 29.1 to 35.7 ppb (mean: 32.4 ppb) | Cardiovascular hospitalizations:  PM2.5: 3043  O_3_: 1357  Respiratory hospitalizations:  PM_2.5_: 455  O_3_: 182 | | |  | **x** |  |  |  |  |  |  |
|  |  |  |  |  |  |  | | | | | |  |  |
| Thongphunchung et al.; 2022 ^67^ | Thailand;  7 years | Exposure:  PM_10_, PM_2.5_  Comparison:  NAAQS (25 µg/m^3^) and 25% of NAAQS (= NAAQS25) | Mortality:  Cardiovascular diseases: PM_2.5_:  NAAQS: 0.2% (0.01; 0.45)  NAAQS25: 3.14% (0.1; 6.99)  Respiratory diseases: PM_2.5_:  NAAQS: 0.2% (0.01; 0.45)  NAAQS25: 3.41% (0.11; 7.66) | | | **x** |  |  |  |  |  |  |  |
|  |  |  |  |  |  |  | | | | | |  |  |
|  |  |  | Mortality:  Cardiovascular diseases: PM_2.5_  NAAQS: 46 (2; 103)  NAAQS25: 718 (23; 1599)  Respiratory diseases: PM_2.5_  NAAQS: 16 (1; 37)  NAAQS25: 278 (9; 624) | | |  | **x** |  |  |  |  |  |  |
|  |  |  |  |  |  |  | | | | | |  |  |
|  |  |  | Outpatient department visits:  Cardiovascular diseases: PM_10_  NAAQS25: 2.83% (0.09; 6.27)  Respiratory diseases: PM_10_  NAAQS25: 2.69% (0.08; 6.06) | | | **x** |  |  |  |  |  |  |  |
|  |  |  |  |  |  |  | | | | | |  |  |
|  |  |  | Outpatient department visits:  Cardiovascular diseases: PM_10_  NAAQS25: 174331 (5544; 386238)  Respiratory diseases: PM_10_  NAAQS25: 143209 (4259; 322618) | | |  | **x** |  |  |  |  |  |  |
|  |  |  |  |  |  |  | | | | | |  |  |
| Thongsame et al.; 2025 ^68^ | Thailand;  4 months | Exposure: PM_2.5_  Comparison: 7.5 µg/m^3^ | All-cause mortality:  20,406 (12,057; 29,613) | | |  |  |  | **x** |  |  |  |  |
|  |  |  |  |  |  |  |  |  |  |  |  |  |  |
| Uda et al.;  2019 ^69^ | Indonesia;  5 years | Exposure: PM_2.5_  Comparison: 10 µg/m^3^ | Mortality:  All-cause: 648 Cardiovascular: 266 \| 119 (adults: ≥ 30y) Respiratory: 55 \| 6 (children: ≤ 5y) Neoplasms: 95 \| 42 (adults: ≥ 30y) | | |  | **x** |  |  |  |  |  |  |
|  |  |  |  |  |  |  | | | | | |  |  |
| Uttajug et al.;  2022 ^70^ | Thailand;  5 years | Exposure:  Total PM_10_ (wildfire-related, including haze days)  Comparison:  Daily average PM_10_ | Respiratory disease-related hospital visits:  Total: 1.3% Haze days: 20.3% | | |  |  | **x** |  |  |  |  |  |
|  |  |  |  |  |  |  | | | | | |  |  |
|  |  |  | Total: 132923 | | |  |  |  | **x** |  |  |  |  |
|  |  |  |  |  |  |  | | | | | |  |  |
| Grosvenor et al.; 2024 ^71^ | Indonesia;  17 years | Exposure: Wildfire-related PM_2.5_  Comparison: 0 µg/m³ | All-cause mortality:  2003-2019: 9,747 (SD: 503.0)  Mean: 573.4 (per year) | | |  | **x** |  |  |  |  |  |  |
|  |  |  |  |  |  |  |  |  |  |  |  |  |  |
| ****Xu et al.;****  ****2024** ^72^** | Southeast Asia (all);  20 years | Exposure: Fire-related PM_2.5_+O_3_  Comparison: 0µg/m³ PM2.5/O3 | All-cause mortality:  206817 (167,202; 246,028) | | |  | **x** |  |  |  |  |  |  |
|  |  |  |  |  |  |  |  |  |  |  |  |  |  |
| Xue et al.;  2021 ^73^ | Southeast Asia (all);  15 years | Exposure:  PM2.5 (wildfire-related)  Comparison: n/A | All-cause mortality:  Indonesia: 11.8% (7.8; 15.7)  Myanmar: 10.2% (6.5; 14.2)  Philippines: 2.7% (-1.4; 6.6) | | | **x** |  |  |  |  |  |  |  |
|  |  |  |  |  |  |  | | | | | |  |  |
|  |  |  | All-cause mortality:  Indonesia: 28876 (19121; 38,371)  Myanmar: 10023 (6418; 14130)  Philippines: 2938 (1499; 7199) | | |  | **x** |  |  |  |  |  |  |
|  |  |  |  |  |  |  | | | | | |  |  |
| Yin; 2022 ^74^ | Cambodia, Laos, Malaysia, Myanmar, Thailand, Vietnam;  30 years | Exposure: PM_2.5_  Comparison: 2.4 µg/m^3^ | All-cause mortality:  1990: 66000 (54000; 77000)  2019: 182000 (150000; 210000) | | |  |  |  | **x** |  |  |  |  |
|  |  |  |  |  |  |  | | | | | |  |  |
| Yin; 2023 ^75^ | Brunei, Indonesia, Malaysia,  Singapore;  30 years | Exposure: PM_2.5_  Comparison: 2.4 µg/m^3^ | All-cause mortality:  1990: 113000 (100000; 125000)  2019: 337000 (300000; 373000) | | |  |  |  | **x** |  |  |  |  |
|  |  |  |  |  |  |  | | | | | |  |  |
| Zhang et al.;  2022 ^76^ | Indonesia, Philippines, Thailand;  20 years | Exposure: PM_2.5_  Comparison: 2.4 to 5.9 µg/m^3^ | All-cause mortality:  16724 | | |  |  |  | **x** |  |  |  |  |
|  |  |  |  |  |  |  |  |  |  |  |  |  |  |
| Zhu et Shi;  2023 ^77^ | Southeast Asia (all);  19 years | Exposure: PM_2.5_  Comparison: 5 µg/m^3^ | All-cause mortality:  SEA total: 3668500  Indonesia: 1784200  Vietnam: 654900  Thailand: 393900  Myanmar: 384500  Philippines: 215400  Malaysia: 125100  Cambodia: 48700  Laos: 30900  Singapore: 30400  Brunei: 500  Yearly deaths:  SEA total: 270140.35  Indonesia: 135512.28  Vietnam: 50135.09  Thailand: 26942.11  Myanmar: 26378.95  Philippines: 14550.88  Malaysia: 8826.32  Cambodia: 3926.32  Laos: 2112.28  Singapore: 1682.46  Brunei: 73.68 | | |  |  |  | **x** |  |  |  |  |
|  |  |  |  |  |  |  | | | | | |  |  |

# Table S3 DLNM extraction

Table S3 provides additional methodological detail for studies with self-established exposure–response functions (n=34), including the modelling approaches used, functional form, DLNM characteristics, and lag structures.

| **Author year** | **Title** | **DLNM usesage** | **Lag period** | **Exposure degrees of freedom (df)** | **Lag df** | **Exposure** |
| --- | --- | --- | --- | --- | --- | --- |
| Aik 2020 | The burden of acute conjunctivitis attributable to ambient particulate matter pollution in Singapore and its exacerbation during South-East Asian haze episodes | No | Lag 0–1weeks in final PM models; lagged exposure effects up to 3 weeks assessed during model building | N/A | N/A | PM2.5, PM10 |
| Cao 2021 | The mortality effect of apparent temperature: A multi-city study in Asia | Yes | 0–21d; sensitivity 15d and 25d | N/A | N/A | Total: Heat + cold  Heat: apparent temperature (temp) > MMT Cold: apparent temp < MMT |
| Chen 2021 | Mortality risk attributable to wildfire-related PM(2·5) pollution: a global time series study in 749 locations | No | Single-day lags 0–7d; main moving average lag 0–2d | N/A | N/A | Wildfire related PM2.5 |
| Chen 2024 | All-cause, cardiovascular, and respiratory mortality and wildfire-related ozone: A multicountry two-stage time series analysis | Yes | 0–2d | N/A | N/A | Wildfire-related O3 |
| Cheng 2020 | Heatwaves and dengue outbreaks in Hanoi, Vietnam: New evidence on early warning | Yes | Lag 0w and lag 1w week for temperature model | N/A | N/A | Heat: temp > 95^th^ |
| Chua 2022 | Projecting Temperature-Attributable Mortality and Hospital Admissions due to Enteric Infections in the Philippines | Yes | Main 0–21d; sensitivity 14d and 27d | 2 | N/A | Non-optimal temp:  temp > 95^th^ + < 5^th^ |
| Dang 2018 | Green Space and Deaths Attributable to the Urban Heat Island Effect in Ho Chi Minh City | Yes | 0–21d | 4 | 5 | Heat (all): temp > 75^th^  Heat (mild): temp between 75^th^-99^th^, Heat (extreme): temp > 99^th^ |
| Denpetkul 2021 | Daily ambient temperature and mortality in Thailand: Estimated effects, attributable risks, and effect modifications by greenness | Yes | 0–21d maximum; key cumulative estimates reported at 0–7d, with cold effects lasting up to 14d | 3 | 5 for all-cause; 3 for cardiovascular; 3 for respiratory | Heat: temp > MMT, Cold: temp < MMT |
| Gasparrini 2015 | Mortality risk attributable to high and low ambient temperature: a multicountry observational study | Yes | 0–21d | N/A | N/A | Heat: temp > MMT, Cold: temp < MMT |
| Huang 2018 | Mortality burden attributable to heatwaves in Thailand: A systematic assessment incorporating evidence-based lag structure | Yes | Acute 0–1d; cumulative optimal lag selected within 1–21d | N/A | N/A | Heatwaves:  2-4 days of temp between:   - mild: 90^th^ - 93^rd^, - medium: 94^th^ - 96^th^,   severe: 96^th^ - 99^th^ |
| Huang 2023 | Global short-term mortality risk and burden associated with tropical cyclones from 1980 to 2019: a multi-country time-series study | Yes | Maximum lag extended to 21d | N/A | 2 | Tropical cyclones  (days with wind speed > 17.5 m/s) |
| Huang 2024 | Risks of infectious disease hospitalisations in the aftermath of tropical cyclones: a multi-country time-series study | Yes | 0–12m; AF/burden over first 2m; sensitivity up to 18m | N/A | N/A | Tropical cyclones  (monthly TC days) |
| Huang 2025 | Hospitalization risk and burden for cause-specific cardiovascular diseases following tropical cyclones: A multicountry study | Yes | 0–12m; cumulative results focused on 6m; sensitivity up to 18m | N/A | N/A | Tropical cyclones  (monthly TC days) |
| Hystad 2020 | Associations of outdoor fine particulate air pollution and cardiovascular disease in 157,436 individuals from 21 countries (PURE): a prospective cohort study | No | N/A | N/A | N/A | Ambient PM2.5 |
| Nguyen 2022 | The protective effect of green space on heat-related respiratory hospitalization among children under 5 years of age in Hanoi, Vietnam | Yes | 0–21d | 4 | 5 | Mild: Heat > 75th < 99th, Extreme: Heat > 99%, Total: > 75th-100th |
| Nguyen 2024 | Effect of ambient air pollution on hospital admission for respiratory diseases in Hanoi children during 2007–2019 | No | Primary lag 0–2d; single-day lags 0–7d explored; AF used lag 0 coefficient | N/A | N/A | PM2.5; NO2 |
| Phung 2021 | Hydropower dams, river drought and health effects: A detection and attribution study in the lower Mekong Delta Region | No | N/A | N/A | N/A | River drought < 5th precentile (77.5cm) |
| Sokharavuth 2023 | Air pollution mitigation assessment to inform Cambodia's first clean air plan | No | N/A | N/A | N/A | PM2.5, NOx, SO2, NH3 |
| Sritong-Aon 2025 | Temporal relationship between ambient temperature and non-accidental mortality in Thailand as evidence of inadequate adaptation to heat | Yes | 0–21d | 3 | 6 | Total: heat + cold,  Heat: temp > MMT,  Cold: temp < MMT |
| Sritong-Aon 2025 | Burden of mortality attributable to diurnal temperature range in Thailand: a nationwide case-crossover analysis from 2007 to 2021 | Yes | Main 0–21d; secondary/sensitivity 0–7d and 0–14d | 3 | 4 | Diurnal temperature range (DTR) |
| Sun 2024 | An estimate of global cardiovascular mortality burden attributable to ambient ozone exposure reveals urban-rural environmental injustice | No | N/A | N/A | N/A | Ambient O3 |
| Tao 2025 | Quantifying localized heatwave impact on mortality: a multi-country modeling study in the Asia-Pacific region | Yes | Up to 2d; largest estimate at lag 0d | 3 | 3 | Heatwave: 3d over the Health-based temperature threshold (Th) (city specific temperature) |
| Tewari 2024 | Associations Between Anthropogenic Factors, Meteorological Factors, and Cause-Specific Emergency Department Admissions | No | 1–3w | N/A | N/A | PM2.5, Temp, Rainfall |
| Thawonmas 2025 | Ambient Temperature and Suicide Risk in Thailand: Evidence from Chiang Mai and Bangkok Provinces | Yes | Main 0–2d; sensitivity 3d, 6d, 10d, 14d | N/A | N/A | Ambient temperature: cool: 0-33rd percentile, warm: 34-66th percentile, hot: 67-100th) |
| Thongphunchung 2022 | Outpatient Department Visits and Mortality with Various Causes Attributable to Ambient Air Pollution in the Eastern Economic Corridor of Thailand | No | Same-day only | N/A | N/A | PM10, PM2.5, NO2, SO2, O3 |
| Tobias 2026 | Variation in reporting of heatstroke mortality: evidence from a multi-country study | Yes | 0–21d | N/A | N/A | Heat > MMT, Extreme heat > 97.5th |
| Vicedo-Cabrera 2021 | The burden of heat-related mortality attributable to recent human-induced climate change | Yes | 0–10d cumulative | N/A | N/A | Heat (4 warmes months) > MMT |
| Wen 2024 | Comparison for the effects of different components of temperature variability on mortality: A multi-country time-series study | No | TV exposures TV0–1d to TV0–7d; daily mean temperature adjustment 0–21d with sensitivity to 28d | N/A | N/A | Temperature variability (interday, intraday) |
| Wen 2024 | Association of cause-specific hospital admissions with high and low temperatures in Thailand: a nationwide time series study | Yes | 0–21d | N/A | N/A | Heat: temp > MMT,  Extreme heat: temp > 97.5^th^,  Cold: temp < MMT,  Extreme cold: temp < 2.5^th^ |
| Wu 2022 | Fluctuating temperature modifies heat-mortality association around the globe | Yes | 0–10d | N/A | N/A | Temperature variability stratified by quantiles |
| Xu 2024 | Global, regional, and national mortality burden attributable to air pollution from landscape fires: a health impact assessment study. | No | 0–2d | N/A | N/A | Landscape fire-sourced PM2.5 + O3 |
| Xue 2021 | Associations between exposure to landscape fire smoke and child mortality in low-income and middle-income countries: a matched case-control study | No | No daily lag assessed | N/A | N/A | PM2.5 |
| Yang 2025 | Hospitalization risks associated with floods in a multi-country study | Yes | 0–21d | Strata function | 4 | Flood days |
| Yang 2026 | Risk, burden, and trend of infectious disease hospitalisations associated with floods: a multicountry, time-series study | Yes | 0–26w | Strata function | 5 | Flood days |

1.	Cao R, Wang Y, Huang J, et al. The Mortality Effect of Apparent Temperature: A Multi-City Study in Asia. *Int J Environ Res Public Health* 2021; **18**(9).

2.	Cheng J, Bambrick H, Yakob L, et al. Heatwaves and dengue outbreaks in Hanoi, Vietnam: New evidence on early warning. *PLOS Neglected Tropical Diseases* 2020; **14**(1): e0007997.

3.	Chua PLC, Ng CFS, Madaniyazi L, et al. Projecting Temperature-Attributable Mortality and Hospital Admissions due to Enteric Infections in the Philippines. *Environmental Health Perspectives* 2022; **130**(2): 027011.

4.	Dang TN, Van DQ, Kusaka H, Seposo XT, Honda Y. Green Space and Deaths Attributable to the Urban Heat Island Effect in Ho Chi Minh City. *Am J Public Health* 2018; **108**(S2): S137-s43.

5.	Denpetkul T, Phosri A. Daily ambient temperature and mortality in Thailand: Estimated effects, attributable risks, and effect modifications by greenness. *Science of The Total Environment* 2021; **791**: 148373.

6.	Gasparrini A, Guo Y, Hashizume M, et al. Mortality risk attributable to high and low ambient temperature: a multicountry observational study. *The Lancet* 2015; **386**(9991): 369-75.

7.	Nguyen VT, Doan QV, Tran NN, et al. The protective effect of green space on heat-related respiratory hospitalization among children under 5 years of age in Hanoi, Vietnam. *Environ Sci Pollut Res Int* 2022; **29**(49): 74197-207.

8.	Sritong-aon C, Phosri A, Sihabut T, Prechthai T. Temporal relationship between ambient temperature and non-accidental mortality in Thailand as evidence of inadequate adaptation to heat. *Ecotoxicology and Environmental Safety* 2025; **299**: 118434.

9.	Thawonmas R, Kim Y, Hashizume M. Ambient Temperature and Suicide Risk in Thailand: Evidence from Chiang Mai and Bangkok Provinces. *Environment & Health* 2025; **3**(5): 560-70.

10.	Tobias A, Honda Y, Madaniyazi L, et al. Variation in reporting of heatstroke mortality: evidence from a multi-country study. *The Lancet Public Health* 2026; **11**(3): e156-e63.

11.	Vicedo-Cabrera AM, Scovronick N, Sera F, et al. The burden of heat-related mortality attributable to recent human-induced climate change. *Nature Climate Change* 2021; **11**(6): 492-500.

12.	Wen B, Kliengchuay W, Suwanmanee S, et al. Association of cause-specific hospital admissions with high and low temperatures in Thailand: a nationwide time series study. *The Lancet Regional Health – Western Pacific* 2024; **46**.

13.	Sritong-Aon C, Phosri A, Sihabut T, Prechthai T. Burden of mortality attributable to diurnal temperature range in Thailand: a nationwide case-crossover analysis from 2007 to 2021. *Trop Med Health* 2025; **53**(1): 78.

14.	Wu Y, Wen B, Li S, et al. Fluctuating temperature modifies heat-mortality association around the globe. *The Innovation* 2022; **3**(2): 100225.

15.	Wen B, Wu Y, Guo Y, et al. Comparison for the effects of different components of temperature variability on mortality: A multi-country time-series study. *Environment International* 2024; **187**: 108712.

16.	Tewari P, Xu B, Pei M, et al. Associations Between Anthropogenic Factors, Meteorological Factors, and Cause-Specific Emergency Department Admissions. *Geohealth* 2024; **8**(9): e2024GH001061.

17.	Huang C, Cheng J, Phung D, Tawatsupa B, Hu W, Xu Z. Mortality burden attributable to heatwaves in Thailand: A systematic assessment incorporating evidence-based lag structure. *Environ Int* 2018; **121**(Pt 1): 41-50.

18.	Huang W, Li S, Vogt T, et al. Global short-term mortality risk and burden associated with tropical cyclones from 1980 to 2019: a multi-country time-series study. *The Lancet Planetary Health* 2023; **7**(8): e694-e705.

19.	Huang W, Vogt T, Park J, et al. Risks of infectious disease hospitalisations in the aftermath of tropical cyclones: a multi-country time-series study. *Lancet Planet Health* 2024; **8**(9): e629-e39.

20.	Huang W, Yang Z, Zhang Y, et al. Hospitalization risk and burden for cause-specific cardiovascular diseases following tropical cyclones: A multicountry study. *Science Advances*; **11**(31): eadr0800.

21.	Phung D, Nguyen-Huy T, Tran NN, et al. Hydropower dams, river drought and health effects: A detection and attribution study in the lower Mekong Delta Region. *Climate Risk Management* 2021; **32**: 100280.

22.	Tao J, Xu Z, Ho HC, et al. Quantifying localized heatwave impact on mortality: a multi-country modeling study in the Asia–Pacific region. *The Lancet Regional Health - Western Pacific* 2025; **61**: 101653.

23.	Yang Z, Huang W, McKenzie JE, et al. Risk, burden, and trend of infectious disease hospitalisations associated with floods: a multicountry, time-series study. *The Lancet Planetary Health* 2026; **10**(1).

24.	Yang Z, Huang W, McKenzie JE, et al. Hospitalization risks associated with floods in a multi-country study. *Nature Water* 2025; **3**(5): 561-70.

25.	Aik J, Chua R, Jamali N, Chee E. The burden of acute conjunctivitis attributable to ambient particulate matter pollution in Singapore and its exacerbation during South-East Asian haze episodes. *Sci Total Environ* 2020; **740**: 140129.

26.	Bui LT, Nguyen NHT, Nguyen PH. Chronic and acute health effects of PM2.5 exposure and the basis of pollution control targets. *Environmental Science and Pollution Research* 2023; **30**(33): 79937-59.

27.	Bui LT, Nguyen PH, My Nguyen DC. Linking air quality, health, and economic effect models for use in air pollution epidemiology studies with uncertain factors. *Atmospheric Pollution Research* 2021; **12**(7): 101118.

28.	Bui LT, Nguyen PH. Evaluation of the annual economic costs associated with PM2.5-based health damage—a case study in Ho Chi Minh City, Vietnam. *Air Quality, Atmosphere & Health* 2023; **16**(3): 415-35.

29.	Chen G, Guo Y, Yue X, et al. Mortality risk attributable to wildfire-related PM<sub>2&#xb7;5</sub> pollution: a global time series study in 749 locations. *The Lancet Planetary Health* 2021; **5**(9): e579-e87.

30.	Chen G, Guo Y, Yue X, et al. All-cause, cardiovascular, and respiratory mortality and wildfire-related ozone: a multicountry two-stage time series analysis. *The Lancet Planetary Health* 2024; **8**(7): e452-e62.

31.	Ha Chi NN, Kim Oanh NT. Photochemical smog modeling of PM2.5 for assessment of associated health impacts in crowded urban area of Southeast Asia. *Environmental Technology & Innovation* 2021; **21**: 101241.

32.	Chowdhury S, Pozzer A, Haines A, et al. Global health burden of ambient PM2.5 and the contribution of anthropogenic black carbon and organic aerosols. *Environment International* 2022; **159**: 107020.

33.	Crippa P, Castruccio S, Archer-Nicholls S, et al. Population exposure to hazardous air quality due to the 2015 fires in Equatorial Asia. *Scientific Reports* 2016; **6**(1): 37074.

34.	Fang T, Gu Y, Li J, Sung JJY, Yim SHL. Effects of climate change on PM2.5 formation chemistry, and the associated human health impacts and economic costs in Southeast Asia. *Environment International* 2025; **205**: 109835.

35.	Fink H, Langselius O, Vignat J, et al. Global and regional cancer burden attributable to modifiable risk factors to inform prevention. *Nature Medicine* 2026; **32**(4): 1306-15.

36.	N RF, Allison MR, B CW, et al. An Assessment of Annual Mortality Attributable to Ambient PM(2.5) in Bangkok, Thailand. *Int J Environ Res Public Health* 2020; **17**(19).

37.	Hein L, Spadaro JV, Ostro B, et al. The health impacts of Indonesian peatland fires. *Environmental Health* 2022; **21**(1): 62.

38.	Hermayurisca F, Taneepanichskul N. Estimation of premature death attributed to short- and long-term PM2.5 exposure in Thailand. *Environmental Monitoring and Assessment* 2023; **195**(10): 1176.

39.	Hu J, Fang T, Yim SHL. Apportioning the ozone-related health impact to precursor emissions and formulating health-oriented ozone control strategies in Southeast Asia. *Journal of Environmental Management* 2026; **401**: 128780.

40.	Hystad P, Larkin A, Rangarajan S, et al. Associations of outdoor fine particulate air pollution and cardiovascular disease in 157 436 individuals from 21 high-income, middle-income, and low-income countries (PURE): a prospective cohort study. *Lancet Planet Health* 2020; **4**(6): e235-e45.

41.	Johnston FH, Henderson SB, Chen Y, et al. Estimated global mortality attributable to smoke from landscape fires. *Environ Health Perspect* 2012; **120**(5): 695-701.

42.	Khempunjakul T, Phosri A, Sangkharat K, Thongphunchung K, Kanchanasuta S, Patthanaissaranukool W. Mortality and economic burden of PM2.5 and NO2 in Thailand using satellite remote sensing and Random Forest algorithms. *Environmental Challenges* 2025; **21**: 101366.

43.	Lai HTN, Nguyen DP, Bui LT. Health and economic impacts of PM2.5 exposure: A case study of pollution-related effects in Hanoi, Vietnam. *Case Studies in Chemical and Environmental Engineering* 2025; **12**: 101296.

44.	Lee HH, Iraqui O, Gu Y, et al. Impacts of air pollutants from fire and non-fire emissions on the regional air quality in Southeast Asia. *Atmos Chem Phys* 2018; **18**(9): 6141-56.

45.	Lelieveld J, Barlas C, Giannadaki D, Pozzer A. Model calculated global, regional and megacity premature mortality due to air pollution. *Atmos Chem Phys* 2013; **13**(14): 7023-37.

46.	Linh Thao NN, Pimonsree S, Prueksakorn K, Bich Thao PT, Vongruang P. Public health and economic impact assessment of PM2.5 from open biomass burning over countries in mainland Southeast Asia during the smog episode. *Atmospheric Pollution Research* 2022; **13**(6): 101418.

47.	Lu H, Xie M, Wang N, et al. The contribution of fires to PM2.5 and population exposure in the Asia Pacific region. *Atmos Chem Phys* 2025; **25**(17): 10141-58.

48.	Marlier ME, DeFries RS, Voulgarakis A, et al. El Niño and health risks from landscape fire emissions in southeast Asia. *Nature Climate Change* 2013; **3**(2): 131-6.

49.	Marlier ME, Liu T, Yu K, et al. Fires, Smoke Exposure, and Public Health: An Integrative Framework to Maximize Health Benefits From Peatland Restoration. *GeoHealth* 2019; **3**(7): 178-89.

50.	Mazeli MI, Pahrol MA, Abdul Shakor AS, Kanniah KD, Omar MA. Cardiovascular, respiratory and all-cause (natural) health endpoint estimation using a spatial approach in Malaysia. *Sci Total Environ* 2023; **874**: 162130.

51.	Mueller W, Vardoulakis S, Steinle S, et al. A health impact assessment of long-term exposure to particulate air pollution in Thailand. *Environmental Research Letters* 2021; **16**(5): 055018.

52.	Nguyen TTN, Vu TD, Vuong NL, et al. Effect of ambient air pollution on hospital admission for respiratory diseases in Hanoi children during 2007-2019. *Environ Res* 2024; **241**: 117633.

53.	Nguyen PH, Nguyen DCM, Bui LT. PM2.5 Pollution Assessment and Quantification of Short-Term Exposure Impacts on Public Health Using a Modeling Approach: A Case Study of Ca Mau Province, Vietnam. In: Boonpook W, Lin Z, Downes NK, Truong QC, editors. Applied Geography and Geoinformatics for Sustainable Development; 2025 2025//; Cham: Springer Nature Switzerland; 2025. p. 265-87.

54.	Nhung NTT, Jegasothy E, Ngan NTK, et al. Mortality Burden due to Exposure to Outdoor Fine Particulate Matter in Hanoi, Vietnam: Health Impact Assessment. *Int J Public Health* 2022; **67**: 1604331.

55.	Park CY, Takahashi K, Fujimori S, et al. Future fire-PM2.5 mortality varies depending on climate and socioeconomic changes. *Environmental Research Letters* 2024; **19**(2): 024003.

56.	Parntrasri W, Puangthongthub S. Weekly extremes of PM2.5-attributable cardiovascular mortality in Northeastern Thailand: Extreme-value return levels and 26-week forecasts. *City and Environment Interactions* 2026; **29**: 100313.

57.	Pinichka C, Bundhamcharoen K, Shibuya K. Diseases Burden of Chronic Obstructive Pulmonary Disease (COPD) Attributable to Ground-Level Ozone in Thailand: Estimates Based on Surface Monitoring Measurements Data. *Glob J Health Sci* 2015; **8**(1): 1-13.

58.	Pinichka C, Makka N, Sukkumnoed D, Chariyalertsak S, Inchai P, Bundhamcharoen K. Burden of disease attributed to ambient air pollution in Thailand: A GIS-based approach. *PLoS One* 2017; **12**(12): e0189909.

59.	Punsompong P, Pani SK, Wang S-H, Bich Pham TT. Assessment of biomass-burning types and transport over Thailand and the associated health risks. *Atmospheric Environment* 2021; **247**: 118176.

60.	Reddington CL, Conibear L, Robinson S, Knote C, Arnold SR, Spracklen DV. Air Pollution From Forest and Vegetation Fires in Southeast Asia Disproportionately Impacts the Poor. *Geohealth* 2021; **5**(9): e2021GH000418.

61.	Sangkham S, Pansakun N, Vongruang P, Hasan MN, Simsin S. Estimated burden of disease and health effects attributable to fine particulate matter and ozone exposure in relation to COPD and cardiovascular disease in Upper Northern Thailand. *Hygiene and Environmental Health Advances* 2026; **17**: 100161.

62.	Shi Y, Matsunaga T, Yamaguchi Y, Zhao A, Li Z, Gu X. Long-term trends and spatial patterns of PM2.5-induced premature mortality in South and Southeast Asia from 1999 to 2014. *Science of The Total Environment* 2018; **631-632**: 1504-14.

63.	Shi Y, Zhao A, Matsunaga T, et al. Underlying causes of PM2.5-induced premature mortality and potential health benefits of air pollution control in South and Southeast Asia from 1999 to 2014. *Environment International* 2018; **121**: 814-23.

64.	Sokharavuth P, Thiv S, Nara C, et al. Air pollution mitigation assessment to inform Cambodia's first clean air plan. *Environmental Research* 2023; **220**: 115230.

65.	Sun HZ, van Daalen KR, Morawska L, et al. An estimate of global cardiovascular mortality burden attributable to ambient ozone exposure reveals urban-rural environmental injustice. *One Earth* 2024; **7**(10): 1803-19.

66.	Syuhada G, Akbar A, Hardiawan D, et al. Impacts of Air Pollution on Health and Cost of Illness in Jakarta, Indonesia. *Int J Environ Res Public Health* 2023; **20**(4).

67.	Thongphunchung K, Charoensuk P, U-tapan S, Loonsamrong W, Phosri A, Mahikul W. Outpatient Department Visits and Mortality with Various Causes Attributable to Ambient Air Pollution in the Eastern Economic Corridor of Thailand. *International Journal of Environmental Research and Public Health*, 2022. (accessed.

68.	Thongsame W, Henze DK, Barth M, et al. Source Attribution and Health Burden of PM2.5 in Mainland Thailand. *GeoHealth* 2025; **9**(9): e2024GH001315.

69.	Uda SK, Hein L, Atmoko D. Assessing the health impacts of peatland fires: a case study for Central Kalimantan, Indonesia. *Environmental Science and Pollution Research* 2019; **26**(30): 31315-27.

70.	Uttajug A, Ueda K, Honda A, Takano H. Estimation of hospital visits for respiratory diseases attributable to PM(10) from vegetation fire smoke and health impacts of regulatory intervention in Upper Northern Thailand. *Sci Rep* 2022; **12**(1): 18515.

71.	Grosvenor MJ, Ardiyani V, Wooster MJ, et al. Catastrophic impact of extreme 2019 Indonesian peatland fires on urban air quality and health. *Communications Earth & Environment* 2024; **5**(1): 649.

72.	Xu R, Ye T, Huang W, et al. Global, regional, and national mortality burden attributable to air pollution from landscape fires: a health impact assessment study. *The Lancet* 2024; **404**(10470): 2447-59.

73.	Xue T, Geng G, Li J, et al. Associations between exposure to landscape fire smoke and child mortality in low-income and middle-income countries: a matched case-control study. *The Lancet Planetary Health* 2021; **5**(9): e588-e98.

74.	Yin S. Decadal changes in premature mortality associated with exposure to outdoor PM2.5 in mainland Southeast Asia and the impacts of biomass burning and anthropogenic emissions. *Science of The Total Environment* 2023; **854**: 158775.

75.	Yin S. Effect of biomass burning on premature mortality associated with long-term exposure to PM(2.5) in Equatorial Asia. *J Environ Manage* 2023; **330**: 117154.

76.	Zhang L, Wilson JP, Zhao N, Zhang W, Wu Y. The dynamics of cardiovascular and respiratory deaths attributed to long-term PM(2.5) exposures in global megacities. *Sci Total Environ* 2022; **842**: 156951.

77.	Zhu Y, Shi Y. Spatio-temporal variations of PM2.5 concentrations and related premature deaths in Asia, Africa, and Europe from 2000 to 2018. *Environmental Impact Assessment Review* 2023; **99**: 107046.
